# Soil–Plant–Animal Mineral Dynamics in Extensive Goat Production Systems: A Critical Review of Geochemical Availability, Soil–Plant Transfer, and Ruminal Bioavailability

**DOI:** 10.3390/ani16172790

**Published:** 2026-09-04

**Authors:** Jesús E. Rivera-Larraga, Fabian E. Olazaran-Santibañez, Arturo Mora-Olivo, Ana G. Reyes, Víctor M. Díaz-Sánchez, Daniel López-Aguirre, Gabriel Aguirre-Guzmán, Cecilia C. Zapata-Campos

**Affiliations:** 1 Facultad de Medicina Veterinaria y Zootecnia, Universidad Autónoma de Tamaulipas, Carretera Victoria-Mante Km 5, Ejido Santa Librada, Ciudad Victoria 87274, Tamaulipas, Mexico; jesus.rivera@uat.edu.mx (J.E.R.-L.); feolazaran@docentes.uat.edu.mx (F.E.O.-S.); gabaguirre@docentes.uat.edu.mx (G.A.-G.); 2 Facultad de Ingeniería y Ciencias, Universidad Autónoma de Tamaulipas, Centro Universitario, Ciudad Victoria 87149, Tamaulipas, Mexico; amorao@docentes.uat.edu.mx (A.M.-O.); dlaguirre@docentes.uat.edu.mx (D.L.-A.); 3 Instituto de Ecología Aplicada, Universidad Autónoma de Tamaulipas, División del Golfo 356, Col. Libertad, Ciudad Victoria 87019, Tamaulipas, Mexico; 4 Programa de Agricultura en Zonas Áridas, Centro de Investigaciones Biológicas del Noroeste, S. C., Av. Instituto Politécnico Nacional 195, Playa Palo Santa Rita Sur, La Paz 23096, Baja California Sur, Mexico; agalvarado@cibnor.mx; 5 Ciencias Pecuarias de Medicina Veterinaria y Zootecnia, Facultad de Estudios Superiores Cuautitlán, Universidad Nacional Autónoma de México, Cuautitlán Izcalli 54714, Estado de Mexico, Mexico; victordiaz@cuautitlan.unam.mx

**Keywords:** *Capra hircus*, mineral nutrition, mineral antagonism, ruminal bioavailability, drylands

## Abstract

Mineral imbalances in extensive goat production are frequently viewed as isolated deficiencies; however, they arise from complex interactions within the soil–plant–animal continuum. This review critically synthesizes evidence on mineral dynamics in goats raised under extensive, arid, and semiarid conditions, with emphasis on geochemical availability, soil–plant transfer, digestive mineral processes, and animal biomarkers. Soil mineral concentrations do not necessarily predict plant uptake or animal mineral status, particularly in alkaline and drought-prone soils where physicochemical factors constrain mineral solubility and availability. Forage plant species play a crucial role in linking soil to goats, although their mineral contributions vary by species, plant part, and season. Furthermore, ruminal physicochemical transformations and mineral antagonisms can alter the amount and chemical form of minerals reaching the intestine, thereby influencing subsequent intestinal absorption and whole-animal utilization, with Cu–Mo–S interactions being particularly relevant. Assessing mineral status in goats requires an integrated approach that considers soil, plant, selected diet, water, supplementation, animal biomarkers, and health outcomes. Future research should prioritize goat-specific predictive studies to improve supplementation strategies and enhance sustainability.

## Simple Summary

Mineral nutrition is vital for the health and productivity of goats, yet imbalances often arise in those raised on natural rangelands. This review examines the flow of minerals through the soil–plant–animal chain in goat production. Factors like soil pH and organic matter affect mineral availability, but goats are selective browsers, complicating predictions of their mineral intake based solely on soil or forage analysis. Absorption can also be hampered by rumen interactions, particularly between copper, molybdenum, and sulfur, potentially causing copper deficiency. Diagnosing imbalances requires a comprehensive approach, integrating soil and water quality, forage composition, supplement intake, and animal biomarkers. The review recommends a framework for identifying mineral risks and emphasizes the need for goat-specific studies and tailored management strategies that consider regional and seasonal conditions.

## 1. Introduction

Extensive goat production systems are widely associated with drylands, rangelands, shrublands, and low-input agroecosystems where animals depend largely on heterogeneous native vegetation. In these systems, goats provide meat and milk and contribute to livelihood security by using forage resources that are often unsuitable for more intensive livestock species. Their adaptive feeding behavior allows them to exploit grasses, forbs, shrubs, tree leaves, pods, flowers, and fruits across variable landscapes and seasons. However, this same heterogeneity makes their mineral nutrition difficult to predict because the mineral composition of the diet depends not only on what is available in the environment but also on what animals select and consume [1,2,3,4,5].

Minerals are required for maintenance, growth, reproduction, immune function, antioxidant defense, skeletal development, rumen metabolism, and productive performance. In extensive systems, mineral imbalances may occur as primary deficiencies due to low dietary supply or as secondary deficiencies caused by antagonistic interactions that reduce absorption or utilization. These imbalances are often subclinical and may not be detected until tissue reserves, immune competence, reproductive efficiency, or productivity are already affected. Seasonal studies in goats from semiarid environments have shown that mineral profiles can vary between dry and rainy periods, underscoring the need to evaluate minerals under field conditions rather than relying solely on generalized recommendations [6]. Recent goat studies also show that mineral disorders may involve functional alterations in hematology, antioxidant status, immunity, and tissue mineral reserves, particularly when trace mineral antagonisms are present [7,8].

Soil is the first component in the mineral pathway, but its relationship with animals’ mineral status is not linear. Total soil mineral concentration does not necessarily represent the fraction available for plant uptake. Geochemical availability is controlled by pH, carbonate content, organic matter, texture, cation exchange capacity, oxides, salinity, and soil moisture. These factors are especially relevant in alkaline, calcareous, arid, and semiarid soils, where micronutrients may be present but poorly available to plants. Dryland studies have shown that micronutrient availability may decrease with increasing pH and may be enhanced by organic carbon, while available phosphorus in calcareous soils can be strongly affected by carbonate content, cation exchange capacity, exchangeable elements, and spatial heterogeneity [9,10,11,12,13]. Therefore, soil analysis for livestock mineral diagnosis should distinguish between total mineral pools and operationally available fractions such as DTPA-extractable micronutrients and Olsen-P.

Forage and browse species constitute the biological interface between soil and goats. However, forage mineral composition is not a passive reflection of soil mineral content. Plant species differ in root architecture, mineral uptake, tissue accumulation, phenological dynamics, and tolerance to edaphic constraints. In semiarid and arid goat systems, shrubs can provide important mineral contributions, but they do not necessarily meet all mineral requirements. Studies in northeastern Mexico and in arid goat production systems have reported that browse species may contain concentrations of some macrominerals considered adequate according to the reference criteria used in the cited studies, whereas phosphorus, copper, zinc, iron, or manganese may fall below those reference values depending on species, season, plant part, and production system [14,15,16,17]. Broader pasture-based evidence also indicates that mineral adequacy cannot be assumed from forage availability alone and should be interpreted in relation to livestock requirements and seasonal variation [18].

A further challenge is that available forage does not match the diet selected by goats. Goats are selective browsers, and their mineral intake depends on the botanical composition and plant parts consumed. In semiarid rangelands, goats may modify intake, diet digestibility, and plant selection according to season and physiological stage, with browse species frequently dominating the diet [1]. Management strategy and drought conditions can also modify diet quality and selection patterns in small ruminants, showing that mineral exposure is shaped by animal behavior as well as by vegetation structure [19,20,21]. This distinction is critical because composite forage samples may overestimate or underestimate mineral intake when they do not represent the selected diet.

Even when minerals are present in the consumed diet, their biological value depends on ruminal physicochemical transformations, subsequent intestinal absorption, and whole-animal utilization. In ruminants, minerals interact with rumen fluid, microbial populations, feed particles, pH conditions, and antagonistic elements before absorption. Trace mineral sources and ruminal solubility influence whether minerals remain available, bind to digesta, become incorporated into microbial biomass, or are transformed into less-absorbable forms. Recent reviews and in vitro studies indicate that inorganic, organic, and hydroxy mineral sources differ in ruminal solubility and potential bioavailability, with important implications for supplementation strategies [22,23,24,25]. Emerging evidence also suggests that dietary copper levels can modify mineral absorbability, rumen microbial composition, and metabolite profiles in grazing small ruminants [26].

Among mineral interactions, the copper–molybdenum–sulfur system is one of the most relevant for grazing ruminants. In the rumen, molybdenum and sulfur can participate in the formation of thiomolybdates, which reduce copper absorption and may induce secondary copper deficiency even when dietary copper appears adequate [27,28,29]. Experimental evidence in Boer-cross goats shows that high molybdenum intake under low-copper conditions can reduce liver copper and iron concentrations and impair immune responses without necessarily causing immediate performance losses [8]. Field evidence in Yudong black goats further demonstrates that copper deficiency may be associated with integrated changes across soil, forage, blood, liver, hematological variables, immune markers, antioxidant status, and response to copper supplementation [7]. These findings illustrate why mineral status cannot be interpreted from dietary concentration alone.

Animal biomarkers provide the most direct evidence of whether environmental and dietary mineral risks translate into biological imbalance. However, the diagnostic value of each biomarker depends on the mineral, matrix, and time scale evaluated. Serum and plasma are practical for herd screening, but they may not reflect long-term tissue reserves for all elements. Liver, bone, hair, whole blood, antioxidant enzymes, immune markers, and productive indicators provide complementary information. Recent work comparing biological substrates in sheep and goats showed that hair should not be considered a direct substitute for blood, plasma, or serum for biomonitoring bioactive elements [30]. Additional goat studies during lactation and under experimentally induced trace mineral imbalances further support the need to interpret mineral biomarkers in relation to physiological stage, tissue reserve dynamics, and functional responses [8,31].

Despite growing evidence in soil science, forage evaluation, ruminant mineral bioavailability, and animal biomonitoring, the literature remains fragmented. Many studies evaluate soil, forage, or animal mineral status separately, whereas fewer integrate geochemical availability, plant transfer, selected diet, ruminal physicochemical transformations, intestinal absorption, and animal biomarkers within a single conceptual framework. Integrated studies in small ruminants and other grazing species indicate that correlations among soil, forage, and animal matrices are element-specific and context-dependent, reinforcing the need for multi-matrix interpretation [32,33,34,35,36,37]. This gap is particularly important in goats because their browsing behavior, seasonal diet shifts, and exposure to heterogeneous vegetation make mineral diagnosis more complex than in systems based on uniform pastures or controlled diets.

Therefore, this review aims to critically synthesize the scientific evidence on soil–plant–animal mineral dynamics in extensive goat production systems, with mechanistic emphasis on mineral systems for which the available evidence is most developed, particularly Cu–Mo–S interactions, P, and Zn, while other macro- and trace elements are incorporated where they contribute relevant evidence on soil–plant transfer, forage mineral composition, animal mineral status, biomonitoring, or system-level interpretation. By integrating evidence from soil science, forage ecology, rumen physiology, animal biomarkers, and production systems, this review proposes a conceptual framework for interpreting mineral imbalances in goats raised under extensive, arid, and semiarid conditions.

## 2. Methodological Approach

### 2.1. Review Design

This study was designed as a critical integrative review supported by a systematized literature search and a narrative synthesis of the evidence. This approach was selected because mineral dynamics in extensive goat production systems involve heterogeneous evidence from soil science, forage ecology, ruminant nutrition, rumen physiology, veterinary medicine, and animal production. The review was not designed as a meta-analysis because the available studies differ substantially in species, production systems, agroecosystems, mineral elements, matrices analyzed, analytical methods, and outcome variables.

The review was guided by the following question: How do soil properties and geochemical mineral availability influence forage mineral composition, ruminant bioavailability, and the mineral, productive, and health status of goats in extensive production systems?

Although this was not a full systematic review, the search, screening, eligibility assessment, and synthesis were informed by PRISMA 2020 reporting principles and PRISMA-S recommendations for transparent search reporting [38,39]. The purpose was to improve methodological transparency while maintaining the flexibility required for an integrative review across disciplines.

### 2.2. Information Sources and Search Strategy

The literature search was conducted in databases relevant to animal science, veterinary medicine, soil science, agronomy, environmental sciences, and rangeland ecology. The main sources were Scopus, Web of Science Core Collection, PubMed, ScienceDirect, CAB Abstracts, SciELO, and Google Scholar. Google Scholar was used as a complementary source to identify open-access studies, regional publications, and relevant articles not retrieved from the main databases.

The principal publication window extended from 1 January 2019 through 29 June 2026. Accordingly, the designation “2019–2026” refers to studies published within this defined period, and 2026 publications were identified through the same structured search framework applied to the preceding years. Older studies were considered only when they provided foundational concepts, standard analytical procedures, classical mineral nutrition principles, or relevant evidence from underrepresented regions or production systems. Searches were conducted in English and Spanish using combinations of free-text terms, Boolean operators, truncation, mineral-specific terms, and production-system descriptors.

Representative search strings are provided were adapted according to the syntax and indexing structure of each database. The final date of the search was 29 June 2026.

### 2.3. Eligibility Criteria

Eligibility criteria are summarized in Table 1. Studies involving goats and small ruminants under extensive or related grazing and browsing conditions were prioritized, whereas evidence from other ruminants was retained only when it provided transferable mechanistic or methodological information on soil–plant–animal transfer, mineral bioavailability, trace mineral antagonisms, or biomonitoring.

### 2.4. Screening and Study Selection

Records were first screened by title and abstract to assess relevance to the review question. Potentially eligible records were then assessed using the full text or a complete online version. All studies included in the final synthesis underwent full-text eligibility assessment; records for which the full text could not be accessed were excluded from the evidence synthesis. Screening considered the study objective, species, production system, geographical context, agroecosystem, mineral elements evaluated, matrices analyzed, analytical methods, and relevance to the soil–plant–animal continuum.

Each article was assigned to one or more thematic domains. When an article overlapped across domains, it was classified according to its primary contribution, while secondary uses were recorded in the extraction matrix. References were also classified by priority according to their direct relevance and applicability to the review question. High-priority studies directly addressed goats or small ruminants, mineral status, grazing or browsing systems, soil–plant–animal transfer, ruminal mineral processes, or mineral-related productive and health outcomes. Medium-priority studies provided relevant but indirect evidence, including studies in other ruminants, controlled feeding trials, or experimental systems with transferable mechanistic information. Low-priority studies were retained only for contextual or methodological support. These priority categories were used to organize the evidence according to relevance and directness and were not intended as a formal risk-of-bias or certainty-of-evidence grading system.

The literature-identification and selection process was summarized using an approximate PRISMA-style flow reconstruction based on the search strings, bibliographic extraction matrix, duplicate-control records, full-text PDF batches, and targeted online verification performed during manuscript development. Overall, 320 records were identified, including 286 records retrieved through database searches and 34 additional records identified through publisher websites, citation tracking, Google Scholar, and targeted online searches. After removing 72 duplicate records, 248 records were screened by title, abstract, and available metadata. Of these, 138 records were excluded because they did not address mineral nutrition, trace elements, soil–plant transfer, ruminant mineral status, ruminal bioavailability, or extensive/semi-extensive production contexts. A total of 110 full-text articles or complete online records were then assessed for eligibility. Thirty-five records were excluded at this stage because they lacked a relevant mineral focus (*n* = 9), were not related to goats, small ruminants, ruminants, soil, plant, or mineral-transfer processes (*n* = 8), were based on intensive or laboratory conditions with limited transferability to extensive systems (*n* = 6), had insufficient methodological detail or unstable source access (*n* = 5), represented duplicate or less complete versions of retained records (*n* = 4), or were outside the final scope except as general background (*n* = 3). The structured eligibility synthesis therefore included 75 references, which were subsequently mapped by thematic domain, species or model, environmental or biological matrix, mineral element, and contribution to the proposed soil–plant–animal conceptual framework. During peer-review revision, two recent goat-specific microbiome studies published before the 29 June 2026 search cutoff were additionally incorporated into Section 5.5 as contextual mechanistic evidence. Because neither study directly evaluated mineral nutrition or a mineral intervention, these two additional references were not incorporated into the 75-reference eligibility matrix or into denominator-based evidence counts.

### 2.5. Data Extraction and Organization

Data were extracted into a structured study-level evidence matrix provided as Supplementary Table S1. For each source, the matrix records bibliographic identification, evidence source class, study design or model, country or region, species or model, species-evidence class, production-system context, environmental or dietary matrices, animal or biological matrices, minerals or elements evaluated, primary thematic domain, main contribution to the review, directness and applicability to extensive goat systems, priority level, synthesis role and double-counting control, denominator status, manuscript sections supported, and an interpretation or limitation note. Evidence-source classification and species-evidence class were used to distinguish direct goat-specific or goat-inclusive evidence from evidence derived from other ruminants, experimental or in vitro models, and soil–plant or methodological studies. When a source contributed to more than one thematic domain, a single primary contribution was assigned for denominator-based accounting, while secondary uses were recorded separately to avoid double counting.

For soil-focused studies, extracted variables included soil type, pH, carbonate content, organic matter, cation exchange capacity, texture, clay fraction, salinity, available phosphorus, DTPA-extractable micronutrients, total mineral concentrations, and spatial or predictive approaches when available. For forage-focused studies, extracted variables included plant species, functional group, plant part, season, phenological stage, mineral composition, and relationship with animal requirements. For animal-focused studies, extracted variables included species, breed or type, age, sex, physiological status, diet, supplementation, biological matrices, mineral concentrations, productive indicators, clinical signs, immune markers, antioxidant markers, and hematological or biochemical variables.

### 2.6. Evidence Synthesis

The evidence was synthesized narratively and critically rather than statistically. The synthesis followed the conceptual sequence of the soil–plant–animal continuum. First, soil properties and geochemical processes regulating mineral availability were reviewed. Second, evidence on mineral uptake by forage, shrubs, browse species, and pasture vegetation was synthesized. Third, the role of goat diet selection and botanical heterogeneity in determining actual mineral intake was evaluated. Fourth, ruminal solubility, reactivity, digesta binding, and mineral antagonisms were analyzed together with evidence of subsequent intestinal absorption and whole-animal bioavailability, with emphasis on copper–molybdenum–sulfur interactions and other relevant trace mineral interactions. Fifth, animal biomarkers of mineral status were evaluated according to diagnostic value, biological interpretation, and matrix-specific limitations. Finally, integrated methodological models combining soil, forage, and animal matrices were assessed to identify research gaps and future directions.

No pooled effect estimates were calculated because the studies were heterogeneous in design, species, mineral elements, matrices, environmental conditions, analytical methods, and outcome variables. Instead, the synthesis emphasized consistency of evidence, mechanistic plausibility, directness of evidence, biological relevance, and applicability to extensive goat production systems. Attention was given to recent studies from arid and semiarid regions and to evidence integrating more than one component of the continuum.

The thematic search strategy used for literature identification is summarized in Table 2.

## 3. Soil Control of Mineral Availability in Extensive Goat Production Systems

### 3.1. Soil Minerals as Potential Supply Rather than Direct Animal Supply

Soil is the first environmental reservoir of mineral elements in extensive goat production systems, but its influence on animal mineral status should not be interpreted as a direct or linear relationship. Total soil mineral concentration provides information about the geochemical background of a site, yet it does not necessarily represent the fraction that is available for plant uptake or, ultimately, for animal utilization. Between soil mineral pools and goat mineral status, there are several sequential filters, including mineral solubility, root uptake, plant accumulation, diet selection, ruminal transformation, intestinal absorption, tissue storage, and physiological regulation.

This distinction is particularly important in arid, semiarid, alkaline, and calcareous environments, where mineral elements may be present in soil but poorly available to vegetation. In such systems, pH, carbonate content, organic matter, clay fraction, cation exchange capacity, oxides, salinity, and soil moisture regulate the mobility and chemical form of nutrients and trace elements [10,11,13]. Therefore, soil-based mineral assessment should distinguish total mineral concentrations from operational soil-test indices such as DTPA-extractable Zn, Cu, Fe, and Mn and Olsen-P. These extraction-based indices estimate specific soil mineral pools under defined analytical conditions and should not be interpreted as universal measures of plant or animal bioavailability; their interpretation depends on soil physicochemical properties, plant species, analytical protocol, and the calibration context in which the soil test is applied [13,40,41,42,43,44].

For goat production systems, this distinction has practical relevance. A soil may contain apparently adequate total concentrations of a given element, while forage plants remain deficient because the available fraction is low. Conversely, a low soil concentration does not always result in low plant concentration if specific species can mobilize or accumulate that element. Thus, soil analysis alone cannot diagnose mineral deficiency in goats, but it provides essential information about the first stage of the soil–plant–animal continuum.

### 3.2. Soil pH, Carbonates, and Micronutrient Availability

Soil pH is one of the main regulators of mineral solubility and plant availability. As pH increases, the availability of several metallic micronutrients, particularly iron, zinc, manganese, and copper, generally decreases due to precipitation, adsorption, or stronger binding to mineral surfaces. This mechanism is especially relevant in calcareous soils, where carbonate-rich conditions maintain alkaline pH and favor chemical reactions that reduce micronutrient mobility [40,41].

Dryland soils frequently combine alkaline pH, low organic matter, irregular moisture, and carbonate accumulation. These characteristics may restrict micronutrient availability even when total soil concentrations are not critically low. Evidence from dryland soils indicates that micronutrient availability is influenced not only by total elemental content but also by organic carbon and soil pH [10]. This is relevant for extensive goat systems because many rangelands and shrublands used by goats are located in environments where high pH and low soil organic matter can reduce the mineral supply available to forage plants.

Carbonate content is also important for phosphorus and zinc dynamics. In calcareous soils, phosphorus can be retained through precipitation with calcium or adsorption onto carbonate-associated surfaces, while zinc availability may be reduced by interactions with carbonates, clay minerals, and iron oxides. Evidence from Mediterranean soils indicates that zinc uptake by plants is affected by carbonate status, clay content, Olsen-P, and oxide composition [40]. This suggests that carbonate-rich soils can generate micronutrient limitations even when total mineral concentrations appear sufficient.

### 3.3. Phosphorus Availability in Alkaline and Calcareous Soils

Phosphorus is a key macromineral for both plant productivity and animal performance, but its availability in soil is strongly controlled by chemical form. In alkaline and calcareous soils, a substantial fraction of phosphorus may be precipitated as calcium phosphates or adsorbed onto mineral surfaces, reducing the amount available for plant uptake. For this reason, Olsen-P can provide a more informative operational index than total phosphorus for estimating potentially plant-accessible soil P in alkaline and calcareous soils; however, its interpretation is soil- and calibration-dependent and should be considered in relation to soil physicochemical properties, plant species, analytical protocol, and locally or regionally validated interpretive ranges rather than as a universal measure of plant P availability [13,42,43].

Recent evidence indicates that phosphorus availability is influenced by organic matter, fertilizer history, carbonate content, exchangeable cations, texture, and spatial heterogeneity. In calcareous soil, the combined use of phosphorus fertilizer and farmyard manure has been shown to increase soil organic matter, available phosphorus, and plant phosphorus uptake [43]. Although this evidence comes from crop systems, the underlying soil processes are relevant to extensive goat systems because similar mechanisms regulate the mineral composition of herbaceous plants, shrubs, and browse species.

Phosphorus availability is also spatially variable. In alkaline calcareous soil, Olsen-P distribution has been associated with carbonate content, cation exchange capacity, exchangeable manganese and zinc, and spatial redistribution processes [13]. In grazing landscapes, this heterogeneity may create mineral patches that affect forage phosphorus concentration and animal exposure. Goats may not use these patches uniformly because their grazing routes, browsing preferences, and seasonal diet composition influence which plant communities are consumed.

### 3.4. Texture, Clay Minerals, Oxides, and Cation Exchange Capacity

Soil texture and mineralogy influence the retention, mobility, and availability of macro- and trace elements. Clay minerals, iron and manganese oxides, organic matter, and exchange sites regulate whether nutrients remain in soil solution, become exchangeable, or are retained in less available forms. These processes are particularly relevant for micronutrients such as zinc, copper, iron, and manganese, which are sensitive to sorption, precipitation, and redox-related transformations.

For zinc, soil–plant transfer is strongly context-dependent. DTPA-extractable zinc is a useful operational indicator, but it does not always predict plant zinc uptake by itself. In calcareous soils, high clay content, carbonate influence, and elevated Olsen-P may reduce plant zinc uptake, while in non-calcareous soils iron oxides can play a stronger role in regulating zinc availability [40]. Studies on phosphorus-induced zinc deficiency also support the need to interpret phosphorus and zinc together rather than as independent soil fertility variables [44].

Cation exchange capacity contributes to nutrient retention by regulating exchangeable pools of calcium, magnesium, potassium, sodium, copper, zinc, manganese, and other cations. Higher cation exchange capacity can improve nutrient retention, but it may also alter mineral ratios and interactions. In calcareous environments, high calcium availability can affect phosphorus chemistry, while exchangeable manganese and zinc may interact with spatial patterns of Olsen-P [13]. Therefore, soil mineral data should be interpreted as part of an interacting chemical system rather than as isolated elemental concentrations.

### 3.5. Organic Matter, Microbial Activity, and Soil Moisture

Organic matter is a central regulator of mineral availability because it affects soil structure, water retention, cation exchange capacity, microbial activity, and complexation of trace elements. In drylands, organic matter is often limited, and this can reduce nutrient cycling and micronutrient availability. Evidence from dryland soils indicates a positive relationship between organic carbon and micronutrient availability, suggesting that soil organic matter may partially buffer the negative effects of aridity and high pH on mineral availability [10].

Microbial activity and rhizosphere processes also influence mineral uptake by plants. Root exudates, organic acids, mycorrhizal associations, and microbial transformations can mobilize phosphorus and trace elements that would otherwise remain poorly available. However, these processes depend strongly on soil moisture. In arid and semiarid systems, irregular rainfall and prolonged dry periods can reduce microbial activity, nutrient diffusion, and root uptake. As a result, mineral transfer from soil to forage may decline during drought, even when extractable mineral pools are present.

For extensive goat production, this has direct implications. During dry periods, plant growth declines, botanical composition changes, and goats often increase browsing of woody species. Thus, soil moisture affects mineral nutrition not only by regulating mineral uptake by plants, but also by modifying the structure of the available diet. This reinforces the need to interpret soil mineral availability together with seasonal forage composition and goat diet selection.

### 3.6. Spatial Heterogeneity and Mineral Patches in Grazing Landscapes

Extensive grazing landscapes are spatially heterogeneous. Parent material, topography, erosion, sediment deposition, shrub cover, manure deposition, grazing pressure, water points, and management history can all generate variation in soil mineral availability. Composite soil samples may obscure this heterogeneity and fail to identify localized areas of mineral deficiency or excess.

In shrublands and semiarid rangelands, vegetation can create fertility islands where organic matter, microbial activity, and nutrient availability differ from open interspaces. Conversely, degraded or eroded areas may show reduced organic matter, lower water retention, and limited nutrient cycling. Because goats move selectively across these heterogeneous landscapes, the average soil mineral value of a paddock may not represent the mineral exposure of the animals.

Recent applications of geostatistics and machine-learning approaches to soil nutrient mapping demonstrate the value of spatial analysis for mineral diagnosis. Predictive models and ordinary kriging have been used to identify spatial variability in Olsen-P and its edaphic drivers [13]. Similar approaches could be adapted to extensive goat systems to identify mineral risk zones, improve sampling design, and support targeted supplementation or grazing management.

### 3.7. Implications for Mineral Diagnosis in Goats

Soil analysis is necessary for identifying edaphic constraints on plant mineral uptake, but it is insufficient to diagnose mineral status in goats directly [10,11,13]. In alkaline and calcareous environments, soil assessment may include pH, carbonate content, organic matter, texture, cation exchange capacity, salinity, and operational soil-test indices such as Olsen-P and DTPA-extractable Zn, Cu, Fe, and Mn; however, these indices should be interpreted according to soil properties, plant species, analytical protocol, and locally or regionally calibrated reference ranges rather than as direct measures of plant or animal bioavailability [10,11,13,40,41,42,43,44]. Spatial heterogeneity and seasonal moisture should also be considered because they influence mineral availability and may obscure localized mineral risk [9,13]. Thus, soil assessment should be interpreted as the first diagnostic stage and used to guide subsequent forage and animal evaluation, particularly when low plant availability or secondary mineral deficiency is suspected [6,7,8]. The main soil factors influencing mineral availability and their relevance for extensive goat production systems are summarized in Table 3.

## 4. Forage Mineral Composition and Soil–Plant Transfer

### 4.1. Forage as the Biological Interface Between Soil and Goats

Forage and browse species constitute the main biological interface between soil mineral availability and goat mineral intake in extensive production systems. Soil properties regulate the potential supply of mineral elements, but plant species determine which fraction of those elements is absorbed, translocated, accumulated in edible tissues, and eventually exposed to grazing or browsing animals. Therefore, forage mineral composition should not be interpreted as a passive reflection of soil mineral content; it is the result of edaphic conditions, plant-specific uptake mechanisms, root architecture, rhizosphere processes, phenological stage, season, and plant functional type [18,19].

This interface is particularly complex in goat systems because goats do not consume vegetation randomly. Unlike production systems based on uniform pastures or formulated diets, extensive goat systems are characterized by heterogeneous vegetation, including grasses, forbs, shrubs, tree leaves, pods, fruits, flowers, and other plant parts. Goats can exploit this heterogeneity through selective browsing, but this also makes mineral intake difficult to predict from average forage samples alone. The mineral profile of the consumed diet may differ substantially from that of the available vegetation, especially in shrublands, rangelands, and drylands [1,2].

The soil–plant transfer of minerals is controlled by both edaphic and botanical factors. Soil pH, organic matter, carbonates, clay minerals, cation exchange capacity, and soil moisture can restrict or facilitate mineral availability, but plant species differ in their capacity to acquire and accumulate minerals under the same soil conditions. Some species may accumulate specific macro- or trace elements, whereas others may maintain low tissue concentrations even when growing in mineral-rich soils [18]. This interspecific variation is especially relevant in rangelands and shrublands, where botanical diversity is high and the diet selected by goats may shift according to season, plant availability, and physiological stage.

### 4.2. Mineral Composition of Shrubs and Browse Species

Shrubs and browse species are central to goat nutrition in arid and semiarid systems, particularly during dry periods when herbaceous biomass declines. Compared with grasses, woody plants may maintain green biomass for longer periods, provide structural and dietary diversity, and contribute minerals that are scarce in seasonal herbaceous forage. However, their mineral contribution is highly variable and depends on plant species, plant part, season, soil conditions, and production system.

Studies in semiarid regions of northeastern Mexico show that shrub species consumed by small ruminants may provide concentrations of calcium, potassium, magnesium, iron, and manganese considered adequate relative to the reference criteria applied in the cited studies, whereas phosphorus, copper, and zinc may fall below those reference values [14,15,17]. This pattern indicates that browse can contribute substantially to macromineral intake, but it may still leave critical trace mineral gaps. In extensive goat systems, these gaps may become more relevant during growth, pregnancy, lactation, or periods of environmental stress, when mineral requirements increase.

Similarly, marked variation has been reported in the mineral content of shrubs from goat production systems in arid zones [16]. In that study, several shrub species provided concentrations of potassium, calcium, magnesium, sodium, boron, chlorine, and nitrogen considered adequate according to the reference criteria used by the authors, whereas phosphorus, zinc, copper, iron, and manganese were frequently below those reference values [16]. These findings support two important principles. First, shrublands cannot be treated as nutritionally uniform systems. Second, mineral supplementation should be based on local forage profiles and seasonal plant composition rather than generalized assumptions about browse quality.

Evidence from broader pasture and rangeland studies reinforces this interpretation. Mineral concentrations in pastures may not consistently meet livestock requirements, and mineral adequacy depends on species composition, soil conditions, season, and animal demand [18]. Although pasture-based evidence is not always goat-specific, it is useful for interpreting the broader variability of mineral supply in forage-based systems.

### 4.3. Seasonal and Phenological Variation in Forage Minerals

Seasonality is one of the main drivers of forage mineral composition in extensive systems. Rainfall distribution, drought intensity, plant growth stage, leaf senescence, biomass availability, and soil moisture can influence both mineral uptake by plants and the relative abundance of plant functional groups. In arid and semiarid regions, mineral concentrations may change between wet and dry seasons due to dilution during rapid growth, concentration during biomass decline, reduced root uptake under drought, or shifts in the species available to animals [45].

In goat systems, seasonal variation in forage mineral composition is further modified by changes in diet selection. During the rainy season, herbaceous plants and forbs may increase in availability, whereas during the dry season goats often depend more heavily on shrubs, woody plants, pods, and persistent browse. Thus, seasonal mineral intake is not determined only by plant physiology but also by animal behavior and vegetation structure [1,4,5].

Seasonal evaluation of mineral status in goats from the semiarid region of Brazil showed that diet mineral composition and animal mineral profiles varied across periods, with low dietary copper, zinc, and cobalt and high iron reported under the conditions studied [6]. Although this study integrated animal matrices, it is also relevant for forage interpretation because it demonstrates that mineral imbalances can persist in native vegetation-based systems and may require region- and season-specific supplementation.

The interaction between season and physiological stage is also important. In semiarid rangelands of Argentina, goats modified feed intake, digestibility, and botanical composition of the diet across seasons and physiological stages, with browse species playing a major role in the selected diet [1]. These findings indicate that forage mineral adequacy should not be assessed only at the vegetation level; it should also consider whether goats are in maintenance, pregnancy, lactation, or growth.

### 4.4. Available Forage Versus Selected Diet

One of the main methodological challenges in mineral diagnosis is the distinction between available forage and selected diet. Available forage refers to the vegetation present in the grazing area, whereas selected diet refers to the specific plant species and plant parts actually consumed by the animals [1,19]. In goats, this distinction is fundamental because selective browsing can substantially modify nutrient and mineral intake.

Composite forage samples may be useful for describing the average mineral composition of a paddock or plant community, but they may not represent what goats consume. A composite sample dominated by grasses may underestimate the importance of shrubs, pods, leaves, or tree species in the diet [20,21]. Conversely, sampling only preferred browse species may overestimate mineral intake if those species are not consumed consistently throughout the season. Therefore, forage mineral evaluation in goat systems should ideally be linked to botanical diet composition, direct observation, bite counts, fecal analysis, or other methods that approximate actual intake.

Diet selection may help goats buffer some mineral limitations by incorporating plant species with higher mineral concentrations. However, selectivity does not guarantee mineral adequacy. Preferred species may be deficient in phosphorus, copper, zinc, or cobalt or may contain secondary compounds that reduce intake, digestibility, or mineral utilization. In addition, drought and grazing pressure may restrict access to mineral-rich plants, forcing animals to consume a narrower and potentially less balanced diet [19,20].

This distinction is especially important when interpreting soil–plant–animal correlations. A weak correlation between forage mineral composition and animal mineral status may not mean that forage is irrelevant; it may indicate that the sampled forage did not represent the selected diet. For goats, the most useful diagnostic approach is to evaluate three levels simultaneously: available vegetation, selected diet, and animal biomarkers [1,19].

### 4.5. Mineral Adequacy and Common Deficiencies in Forage-Based Systems

Forage mineral adequacy should be interpreted in relation to animal requirements, but these requirements vary according to age, breed, physiological stage, productivity, health status, and environmental stress. A forage resource that is adequate for maintenance may be insufficient for late pregnancy, early lactation, growth, or immune challenge. Therefore, mineral thresholds should be applied cautiously and interpreted within the productive context [45,46,47].

Several studies across diverse semiarid grazing environments indicate that phosphorus, copper, zinc, cobalt, and sometimes selenium are among the minerals most likely to be limiting in forage-based small-ruminant systems [6,18]. In contrast, calcium, potassium, magnesium, iron, and manganese may occur at comparatively higher concentrations, although their levels also vary with plant species, soil background, and season [14,16]. This pattern is important because it suggests that forage-based diets may provide sufficient amounts of some minerals while simultaneously failing to meet requirements for others.

Representative field data illustrate the magnitude of these mineral limitations. In semiarid northeastern Mexico, the annual mean foliar concentrations of five shrub species consumed by small ruminants were 1.3 g P/kg DM, 6.4 mg Cu/kg DM, and 17.7 mg Zn/kg DM [14]. In arid goat-production systems in Baja California Sur, mean shrub concentrations across extensive, intensive, and semi-intensive systems ranged from 1.61 to 2.29 g P/kg DM, 4.20 to 4.68 mg Cu/kg DM, and 2.67 to 3.17 mg Zn/kg DM [16]. These values provide quantitative examples of the magnitude of P, Cu, and Zn limitations reported in extensive and semiarid forage systems [14,16].

For practical interpretation, these concentrations should be compared with requirements for a defined physiological state. Goat-specific mineral requirements vary according to body weight, dry-matter intake, milk yield, and physiological stage, with greater mineral demands associated with productive states such as lactation [46]. Therefore, forage concentrations should not be classified simply as adequate or deficient without considering the animal category, expected intake, mineral bioavailability, and antagonistic interactions [45,46,48,49].

### 4.6. Soil–Plant Transfer and Diagnostic Limitations

Soil–plant mineral transfer is mineral-specific, soil-specific, and plant-specific. Some elements may show a stronger relationship between soil availability and plant concentration, whereas others are strongly regulated by plant uptake mechanisms, rhizosphere processes, or antagonistic interactions in the soil. For example, zinc uptake may be constrained by carbonate content, clay fraction, iron oxides, and high available phosphorus, while phosphorus availability may be limited by calcium carbonate and low organic matter in alkaline soils [13,40].

These mechanisms create important diagnostic limitations. Low mineral concentration in soil does not always produce low mineral concentration in forage if plants can mobilize or accumulate that element [18]. Conversely, a soil with apparently adequate total mineral concentrations may still produce mineral-deficient forage when the available fraction is low. Therefore, soil and forage analyses should be interpreted jointly, with attention to the chemical form of the mineral and the botanical identity of the forage species.

Forage analysis is closer to animal intake than soil analysis, but it still does not define actual mineral intake in selective browsers. In extensive goat systems, forage sampling should consider the main species consumed, the plant parts selected, and the season of use. Leaves, stems, pods, flowers, fruits, and young shoots can differ substantially in mineral concentration [14]. Sampling the wrong plant fraction may lead to incorrect conclusions about mineral adequacy.

A stronger diagnostic strategy should therefore combine soil availability indicators, species-specific forage mineral analysis, botanical diet composition, and animal biomarkers [1,45]. This approach can help distinguish whether a mineral problem originates from low soil availability, poor plant uptake, low concentration in preferred species, restricted access to mineral-rich plants, antagonistic interactions, or increased animal demand.

### 4.7. Implications for Mineral Management in Extensive Goat Systems

Forage mineral composition is a key but variable link between soil and animal mineral status. Browse species can provide important mineral contributions in drylands but do not ensure balanced nutrition; P, Cu, Zn, Co, and sometimes Se may be limiting, while high Fe, S, or Mo can contribute to secondary deficiencies through reduced mineral bioavailability [6,18,45,50,51].

Mineral management should therefore rely on local and seasonal evidence rather than generalized supplementation programs, integrating forage and selected-diet information with relevant soil, water, and animal indicators. This is particularly important in arid and semiarid systems, where vegetation composition and mineral intake can change markedly between wet and dry seasons [6]. The key forage-related factors influencing mineral intake in extensive goat systems are summarized in Table 4.

## 5. Ruminal Solubility, Antagonisms, and Mineral Bioavailability

### 5.1. Dietary Mineral Concentration Versus Biological Availability

The mineral concentration of forage, selected diet, or supplement represents only the potential mineral supply available to grazing goats. It does not necessarily indicate the fraction that will be solubilized, absorbed, transported, stored, or used in physiological processes. In ruminants, this distinction is especially important because minerals are exposed to ruminal fermentation, microbial metabolism, variable pH, redox conditions, digesta binding, and antagonistic elements before intestinal absorption occurs [22,48,49].

In this review, ruminal solubility and reactivity refer specifically to physicochemical events occurring within the rumen, including dissolution, precipitation, complex formation, microbial incorporation, and binding to digesta or antagonistic compounds. These processes determine the chemical form and amount of mineral reaching the lower gastrointestinal tract but do not, by themselves, demonstrate intestinal absorption or whole-animal bioavailability [23,24,52,53]. Intestinal absorption refers to the transfer of mineral across the gastrointestinal epithelium into the animal, whereas relative bioavailability refers to the comparative extent to which an ingested mineral source is absorbed and utilized by the animal relative to an appropriate reference source, as evaluated through animal-level responses or validated status indicators [22,48,49]. Accordingly, ruminal solubility or in vitro ruminal reactivity should not be interpreted as synonymous with relative bioavailability unless subsequent absorption or utilization has been demonstrated.

Therefore, mineral nutrition in goats should not be interpreted exclusively from total dietary concentrations. A diet may contain an apparently adequate concentration of a given element, but the animal may still develop deficiency if the mineral is poorly soluble, chemically unavailable, strongly bound to digesta, or antagonized by other dietary components. Conversely, marginal dietary concentrations may not result in deficiency when the mineral source is highly available, when tissue reserves are adequate, or when animals compensate through selective feeding and supplementation [22,54].

This principle is central to the soil–plant–animal continuum. Soil properties influence forage mineral composition, and forage determines potential mineral intake, but the transition from ingested mineral to biologically available mineral involves distinct digestive and physiological processes. Ruminal solubility, reactivity, digesta binding, microbial incorporation, and antagonistic transformations determine the chemical forms and quantities of minerals that reach the lower gastrointestinal tract, whereas intestinal absorption determines their entry into the animal and contributes to subsequent whole-animal bioavailability and utilization [22,23,48,49]. Thus, interpretation of mineral risk in extensive goat systems requires integration of dietary concentration, mineral source, ruminal physicochemical behavior, intestinal absorption, antagonistic interactions, physiological demand, and animal biomarkers.

### 5.2. Mineral Source, Ruminal Solubility, and Digesta Binding

Trace minerals differ in ruminal solubility according to their chemical form. Inorganic sources such as sulfates, oxides, carbonates, and chlorides may behave differently from organic, chelated, hydroxy, or protected forms. These differences influence whether minerals dissociate in ruminal fluid, bind to feed particles, interact with antagonists, become associated with rumen microorganisms, or pass to the lower gastrointestinal tract [23,25,52].

The source of copper, zinc, and manganese can modify mineral solubility and distribution within ruminal fractions. Trace mineral source has been shown to influence the ruminal distribution of copper and zinc and their binding strength to ruminal digesta, supporting the idea that ruminal behavior is not determined only by total mineral concentration [53]. Additional evidence indicates that trace mineral source can affect rumen trace mineral metabolism, fiber digestion, ruminal fermentation, and the distribution of copper, zinc, and manganese under different dietary models [55,56]. These findings indicate that mineral form can influence both mineral metabolism and rumen function.

In vitro evidence also supports source-dependent differences. Inorganic manganese, zinc, and copper sources show different ruminal solubility patterns, with sulfate forms generally being more soluble than oxide forms [24]. However, solubility does not always translate directly into biological availability or favorable fermentation responses. These findings reinforce that less soluble mineral forms may reduce reactive interactions in the rumen compared with highly soluble inorganic salts, whereas high solubility does not necessarily imply more efficient animal utilization [22,24].

For extensive goat systems, these findings imply that mineral supplementation should consider not only the concentration of each element but also its chemical source, ruminal solubility and reactivity, supplement intake, basal forage composition, and antagonistic context. The same nominal amount of copper, zinc, or manganese may undergo different physicochemical transformations in the rumen, including dissolution, precipitation, complex formation, microbial association, or binding to digesta, thereby altering the amount and chemical form of mineral reaching the lower gastrointestinal tract [22,48]. However, these ruminal processes should not be interpreted as direct measures of intestinal absorption or whole-animal bioavailability unless subsequent absorption or utilization has been demonstrated.

### 5.3. Copper–Molybdenum–Sulfur Interaction

The copper–molybdenum–sulfur interaction is one of the most important mechanisms of secondary mineral deficiency in ruminants. Copper is required for iron metabolism, antioxidant defense, connective tissue formation, pigmentation, immune function, and multiple enzymatic systems. However, its absorption can be markedly reduced when molybdenum and sulfur are present at elevated concentrations in the diet [27,28,29,49].

In the rumen, molybdate can react with sulfide derived from dietary sulfur to form thiomolybdates. These compounds bind copper and reduce its availability for absorption, potentially inducing secondary copper deficiency even when total dietary copper appears adequate. This mechanism is particularly relevant in grazing and browsing systems because soil, forage, water, supplements, and soil ingestion may all contribute variable concentrations of copper, molybdenum, sulfur, and iron [27,57].

Recent studies continue to show that copper bioavailability is strongly modified by antagonistic conditions. The relative bioavailability of organic bis-glycinate-bound copper and inorganic copper sulfate has been evaluated in beef steers fed a high-antagonist diet, reinforcing that mineral source can influence copper availability when molybdenum, sulfur, or other antagonists are present [58]. Likewise, copper solubility under rumen-simulated conditions can be affected by the copper source and by the presence of iron and sulfur [59].

Goat-specific evidence also supports the biological importance of copper antagonisms [7,8]. In Boer-cross goats fed low-copper diets, increasing dietary molybdenum reduced liver copper and iron concentrations and impaired immune responses, even without strong short-term effects on growth performance [8]. Likewise, copper-deficient Yudong black goats showed low copper concentrations in blood and liver, high sulfur exposure, hematological alterations, impaired immune and antioxidant status, and improvement after copper sulfate treatment [7]. These findings indicate that copper deficiency in goats may arise from environmental and ruminal antagonisms rather than from low dietary copper alone.

### 5.4. Other Mineral Interactions Relevant to Extensive Systems

Although Cu–Mo–S is the best-known antagonistic system, other mineral interactions are also relevant in extensive goat production. High dietary iron may interfere with copper metabolism, especially when copper intake is marginal or when molybdenum and sulfur are also elevated. This is important in semiarid forage-based systems where high iron intake may coexist with low dietary copper, zinc, or cobalt, increasing the risk of multiple trace mineral imbalances [6,49].

Zinc and copper should also be interpreted together because both participate in enzyme systems, antioxidant defense, immune function, epithelial integrity, reproduction, and growth. Excessive supplementation or imbalance of one element may affect the metabolism or status of the other. Studies on zinc supplementation and ruminal fermentation indicate that zinc form and concentration can influence microbial activity, digestibility, and ruminal function, suggesting that zinc should not be evaluated only as a dietary concentration [60,61].

Calcium and phosphorus interactions are also central to mineral diagnosis. Both minerals are required for skeletal development, neuromuscular function, metabolism, and productivity, but their biological value depends partly on dietary balance. In shrub-based systems, browse species may contain relatively high calcium concentrations while phosphorus remains comparatively low, potentially creating a mineral imbalance that can affect growth, reproduction, and skeletal mineralization if not corrected through targeted supplementation [14,16].

Sulfur deserves specific attention because it can reduce copper availability even when molybdenum is not extremely high. Sulfur sources may include forage, drinking water, soil ingestion, by-products, and mineral supplements. Therefore, unexplained copper deficiency in grazing goats should not be evaluated only through copper concentration; sulfur, molybdenum, iron, water mineral composition, and liver copper should also be considered [27,62].

### 5.5. Rumen Microbiota, Fermentation, and Mineral Utilization

Trace minerals are not only absorbed by the animal; they also interact with rumen microorganisms. Copper, zinc, manganese, iron, cobalt, and selenium participate in microbial enzyme systems, fiber degradation, fermentation, microbial growth, and redox metabolism. However, rumen microbial responses depend on mineral concentration, chemical source, solubility, basal diet, and tolerance thresholds. Deficient mineral supply may impair microbial metabolism, whereas excessive soluble minerals may inhibit specific microbial populations or alter fermentation patterns [49].

Recent goat-specific studies, added during peer-review revision as contextual mechanistic evidence and excluded from the 75-reference structured eligibility denominator, provide complementary evidence that microbial ecology can be linked to fermentation and systemic host responses, although these studies did not directly evaluate mineral interventions. In Yudong black goats, dietary cecropin supplementation modified rumen fermentation and microbiota while improving growth performance, serum antioxidant capacity, and immune indices [63]. In a separate goat study, *Enterococcus faecalis* JM263 supplementation under a high-concentrate diet remodeled the gut microbiota, improved intestinal barrier and immune responses, and attenuated hepatic oxidative stress through changes associated with Nrf2/Keap1 signaling and arachidonic acid metabolism [64]. Accordingly, these studies should be interpreted as contextual mechanistic evidence supporting microbiota–host interactions rather than as direct evidence that mineral supplementation drives these responses.

Recent research has increased interest in the effects of trace minerals on rumen fermentation and microbial ecology. Lipid microencapsulation of copper sulfate and sodium selenite has been shown to modify ruminal microbial fermentation in a dual-flow continuous-culture system [65]. In vitro evidence also indicates that copper level can influence ruminal fermentation, bacterial growth, and methane production, demonstrating that copper can affect rumen microbial activity beyond its role as an absorbed nutrient [66].

Mineral source and protection technologies can also modify ruminal outcomes. Copper sulfate and coated copper sulfate have been shown to affect nutrient digestibility, ruminal fermentation, and blood metabolites in dairy cows [61], whereas encapsulated copper can influence in vitro rumen fermentation and methane production [67]. These findings suggest that protected or encapsulated mineral sources may alter ruminal reactivity and microbial exposure compared with unprotected inorganic salts.

Evidence from small ruminants and related models also supports this interpretation. In growing sheep, coated and uncoated trace elements have been shown to differ in their effects on growth performance, apparent digestibility, intestinal development, and microbial diversity [68]. In goats and sheep fed high-fiber tannin-rich diets, copper, iron, zinc, and tannin concentrations varied throughout the digestive tract, reinforcing the importance of considering mineral interactions within the digestive environment rather than only at the dietary level [69].

### 5.6. Implications for Interpreting Forage and Supplement Mineral Data

Forage and supplement analyses should be interpreted as estimates of potential mineral supply rather than definitive evidence of animal mineral status, because the biological response depends on actual intake, mineral source, ruminal solubility, antagonistic exposure, water mineral content, physiological demand, and tissue reserves [22,54]. In extensive goat systems, diverse and seasonally changing diets further complicate this interpretation. Low Cu status may reflect inadequate forage Cu, poor supplement intake, or exposure to Mo, S, and Fe [7,27], whereas low Zn status may result from low forage Zn, edaphic constraints on plant uptake, dietary interactions, or inadequate supplementation [16,40]. Accordingly, forage and supplement data should be interpreted together with selected-diet information and appropriate animal biomarkers [30,54].

### 5.7. Practical Implications for Extensive Goat Production

Mineral supplementation in extensive goat systems should be targeted, region-specific, and responsive to seasonal and physiological changes. When Cu deficiency is suspected, assessment should include Mo, S, Fe, water composition, and liver Cu rather than Cu concentration alone [8,62]. Likewise, suspected P deficiency should be evaluated using forage P, Ca:P ratio, soil Olsen-P, and relevant animal indicators, particularly in arid and semiarid systems where browse may supply adequate Ca but insufficient P [16,43]. Supplementation strategies should also account for seasonal shifts in diet composition and the increased mineral demands associated with growth, pregnancy, and lactation [1,6]. Ruminal solubility, reactivity, digesta binding, and antagonistic interactions should therefore be considered separately from intestinal absorption and whole-animal mineral utilization when interpreting mineral status and designing supplementation programs [22,48,49]. The main mineral interactions relevant to extensive goat production systems are summarized in Table 5.

## 6. Animal Biomarkers of Mineral Status in Goats

### 6.1. The Animal as the Final Integrator of Mineral Exposure

Animal biomarkers are essential for interpreting mineral status in extensive goat production systems because they reflect the final biological outcome of soil mineral availability, forage mineral composition, selected diet, water intake, supplementation, ruminal physicochemical transformations, intestinal absorption, antagonistic interactions, physiological demand, and homeostatic regulation. While soil and forage analyses describe potential mineral supply, animal-based indicators help determine whether minerals are actually absorbed, transported, stored, mobilized, or functionally used [30,54,70,71].

In the soil–plant–animal continuum, the animal represents the integrative diagnostic level. A mineral deficiency detected in goats may originate from low soil availability, low forage concentration, poor intake of mineral-rich plant species, low supplement consumption, unfavorable ruminal physicochemical transformations, antagonistic inhibition of intestinal absorption, or increased physiological demand. Conversely, low mineral concentration in forage does not always result in animal deficiency if goats compensate through selective browsing, tissue reserves, or supplementation [6,72].

However, no single biomarker can fully describe mineral status for all elements. Different minerals are regulated by different physiological mechanisms, and biological matrices reflect different time scales of exposure. Blood-based indicators may reflect short-term circulating status, liver may reflect tissue reserves for some trace elements, bone may reflect longer-term macromineral storage, and hair may provide complementary information but is influenced by external contamination, pigmentation, sampling site, and environmental exposure [30,54,70].

### 6.2. Blood, Serum, and Plasma Indicators

Serum and plasma are among the most frequently used matrices for evaluating mineral status in goats and other small ruminants because they are practical, relatively non-invasive, and useful for herd-level screening. They can provide information on circulating concentrations of calcium, phosphorus, magnesium, sodium, potassium, copper, zinc, iron, selenium, and other elements, particularly when interpreted together with diet, season, physiological stage, and clinical status [31,54,71].

Nevertheless, circulating mineral concentrations are not always direct indicators of total body reserves. Some minerals are tightly homeostatically regulated, so serum or plasma values may remain within reference intervals until tissue reserves are depleted or deficiency becomes clinically relevant. This is particularly important for copper, calcium, phosphorus, and zinc, where circulating concentrations may not fully reflect long-term nutritional status or tissue depletion [54,70].

Recent goat-specific studies provide quantitative examples of the value of blood-based monitoring. In goats raised on native pasture in the Brazilian semiarid region, serum Co concentrations in unsupplemented animals ranged from 0.11 ± 0.04 to 0.50 ± 0.20 µmol/L across seasonal periods; the lowest seasonal mean was below the 0.17–0.51 µmol/L reference interval used in that study [6]. In goat kids, serum Se concentrations were 97.6 ± 3.5 and 93.1 ± 3.5 ng/mL in control animals compared with 81.9 ± 3.5 and 71.3 ± 3.5 ng/mL in sericea lespedeza-supplemented animals in 2012 and 2013, respectively [51]. These findings illustrate that quantitative interpretation of Co and Se status should consider season, dietary treatment, and the biological matrix evaluated [6,51].

Comparative matrix studies also indicate that serum, plasma, and whole blood should not be considered interchangeable. In sheep and goats, calcium, copper, iron, magnesium, manganese, sodium, and zinc have been evaluated in whole blood, serum, blood clot, plasma, plasma sediment, and hair, with differences in element biodistribution reported among substrates and between species [30]. This reinforces that matrix selection can influence diagnostic interpretation and that biomarker equivalence should not be assumed.

### 6.3. Liver and Tissue Reserves

Liver mineral concentration is particularly important for assessing body reserves of selected trace elements, especially copper. In ruminants, liver copper is generally more informative than serum copper for evaluating long-term copper status because circulating copper may remain relatively stable until hepatic reserves are substantially depleted. Therefore, liver analysis can detect subclinical copper depletion before severe clinical signs or obvious production losses appear [54,70]. Goat studies involving copper antagonism illustrate the diagnostic importance of liver mineral evaluation. In Boer-cross goats fed low-copper diets, increasing dietary molybdenum reduced liver copper and iron concentrations and impaired immune responses, even though growth performance and some blood metabolites were not strongly affected during the experimental period [8]. This suggests that tissue reserves and functional indicators may reveal early biological effects of mineral imbalance before visible production losses are detected.

Field evidence also supports the value of combining liver and blood indicators. In copper-deficient Yudong black goats, a multi-matrix assessment incorporating soil, forage, blood, liver, hematological, biochemical, immune, antioxidant, and treatment-response variables linked low blood and liver copper with altered hematology, reduced ceruloplasmin, impaired immune function, oxidative stress, and improvement after copper sulfate treatment [7]. This type of multi-matrix diagnosis is especially useful when deficiency is secondary to antagonistic interactions rather than simple dietary shortage.

Bone and rib mineral analyses may be useful for evaluating longer-term macromineral status, particularly calcium and phosphorus. Bone mineralization reflects chronic mineral supply more directly than serum concentrations for some conditions, although sampling is more invasive and less practical under field conditions. Seasonal goat studies that include diet, liver, and rib indicators support the value of tissue-based interpretation when serum alone is insufficient [6,70].

### 6.4. Hair and Other Non-Invasive Matrices

Hair is attractive as a biomonitoring matrix because it is easy to collect, non-invasive, stable during storage, and potentially useful for evaluating longer-term exposure. However, its interpretation is complex because mineral concentrations in hair may be affected by external contamination, dust, soil contact, washing procedures, pigmentation, growth rate, body sampling site, age, breed, physiological status, and environmental exposure [30,70,73,74].

Hair should not be considered a valid alternative to blood, plasma, or serum for biomonitoring bioactive elements in sheep and goats [30]. This does not mean that hair analysis lacks value, but it should be interpreted as a complementary matrix rather than as a substitute for blood-based or tissue-based indicators. In extensive systems, this caution is particularly important because goats are frequently exposed to soil, dust, shrubs, mineral particles, and environmental contaminants [30].

Other non-invasive or minimally invasive matrices may also contribute to biomonitoring, but their diagnostic value must be validated for each mineral. In Kilis goats, macro- and trace-element concentrations have been evaluated in serum, tears, saliva, and hair, illustrating the growing interest in alternative matrices [73]. However, such matrices should not be adopted as primary diagnostic tools until their relationship with established biomarkers, tissue reserves, and physiological outcomes is clearly defined.

Hair-related indicators may complement mineral diagnosis when interpreted alongside blood, serum, plasma, or tissue matrices. However, because hair mineral concentrations may reflect both internal status and environmental contamination, hair should be treated as a secondary biomonitoring matrix rather than a standalone diagnostic substitute [30,73].

### 6.5. Functional Biomarkers: Hematology, Immunity, and Oxidative Status

Mineral status should not be evaluated only through mineral concentrations. Functional biomarkers are necessary to determine whether mineral imbalances have physiological consequences. Trace elements such as copper, zinc, selenium, iron, manganese, and cobalt participate in enzyme systems, antioxidant defense, erythropoiesis, immune function, endocrine regulation, connective tissue formation, and cellular metabolism [49,54].

Copper deficiency provides a clear example of the need for functional interpretation. Copper is involved in iron metabolism through ceruloplasmin, antioxidant defense through copper-zinc superoxide dismutase, immune function, pigmentation, connective tissue metabolism, and nervous system function. Therefore, copper deficiency may be reflected not only in low tissue copper but also in anemia-related variables, reduced ceruloplasmin, impaired immune response, and oxidative stress [7,8,49].

Copper-deficient goats have shown reduced hemoglobin-related variables, reduced ceruloplasmin, lower immunoglobulin concentrations, decreased antioxidant enzyme activity, and increased oxidative stress indicators [7]. In addition, high dietary molybdenum has been associated with reduced liver copper concentrations and impaired immune responses in Boer-cross goats [8]. These findings indicate that functional biomarkers can reveal subclinical biological effects even when performance responses are not yet evident.

Selenium status is another example where functional biomarkers can be important. Serum selenium concentration may be useful, but glutathione peroxidase activity can provide additional information on selenium-dependent antioxidant function. Therefore, selenium biomarkers should be interpreted at the herd level and in relation to diet, region, physiological stage, and management rather than as isolated values [49,54].

### 6.6. Productive and Reproductive Indicators

Mineral imbalances can affect growth, reproduction, milk production, kid survival, immune competence, disease resistance, and general herd productivity. However, productive and reproductive indicators are usually nonspecific and may appear only after mineral imbalance has persisted for a considerable time. Poor growth, low body condition, reproductive failure, weak offspring, reduced milk yield, or increased disease susceptibility may be associated with mineral deficiency, but they can also result from energy deficiency, protein limitation, parasitism, infectious disease, heat stress, or management factors [54,72].

For this reason, productive and reproductive variables should be interpreted as biological outcomes rather than primary diagnostic tools. They are useful for assessing the practical relevance of mineral imbalances, but they cannot identify the specific mineral involved without complementary laboratory analyses. For example, poor growth or reproductive performance in extensive goat systems may be related to phosphorus, copper, zinc, selenium, cobalt, or multiple deficiencies, but diagnosis requires integration of forage, water, supplement, and animal biomarker data [6,54].

Physiological stage is particularly important in goats because mineral demand changes during growth, pregnancy, and lactation. Mineral, metabolic, and oxidative profiles have been shown to vary in Saanen goats under lactation conditions [75], while blood mineral profiles have also been characterized in French Alpine goats during the first third of lactation [31]. These findings support the need to interpret mineral biomarkers according to production stage rather than using a single universal threshold.

### 6.7. Interpretation of Biomarkers in Extensive Systems

Biomarker interpretation in extensive goat systems is influenced by heterogeneous diets, seasonal forage variation, supplement intake, water mineral composition, physiological stage, health status, and environmental conditions. Consequently, reference values derived under controlled conditions may not always be directly applicable to grazing or browsing goats, and breed, age, sex, and management should also be considered [6,54,72,76].

Biomarkers also differ in diagnostic sensitivity and specificity. Serum Cu may be less sensitive than liver Cu during early depletion, serum P may vary with dietary supply and homeostatic regulation, and hair may reflect both endogenous status and environmental exposure [30,54,70]. Therefore, biomarker selection should be mineral-specific and, when deficiency or antagonism is suspected, complementary matrices should be used to distinguish primary deficiency, secondary deficiency, inadequate intake, and increased physiological demand [6,7].

### 6.8. Implications for Research and Diagnosis

Future research on mineral status in extensively managed goats should prioritize longitudinal, multi-matrix designs that account for seasonal variation. Single-time-point serum measurements or forage analyses alone may fail to detect tissue reserve depletion, compensatory dietary responses, or antagonistic interactions [30,54]. Evidence from semiarid goat systems indicates that concurrent evaluation of diet, serum, liver, and rib mineral status across seasons provides a more comprehensive assessment of mineral dynamics [6]. Accordingly, complementary matrices should be selected according to the mineral, production context, and specific diagnostic or research question. The main animal biomarkers for evaluating mineral status in goats are summarized in Table 6.

## 7. Integrated Soil–Plant–Animal Framework for Mineral Diagnosis in Extensive Goat Systems

### 7.1. Rationale for an Integrated Diagnostic Framework

Mineral diagnosis in extensive goat production systems requires an integrated framework because no single environmental, dietary, or animal matrix can explain mineral status independently. Soil geochemical availability, forage mineral composition, selected diet, ruminal physicochemical transformations, intestinal absorption, and animal response represent sequential but non-linear levels of mineral transfer, and discordance among these levels should therefore be expected [34,37].

**Figure 1:**
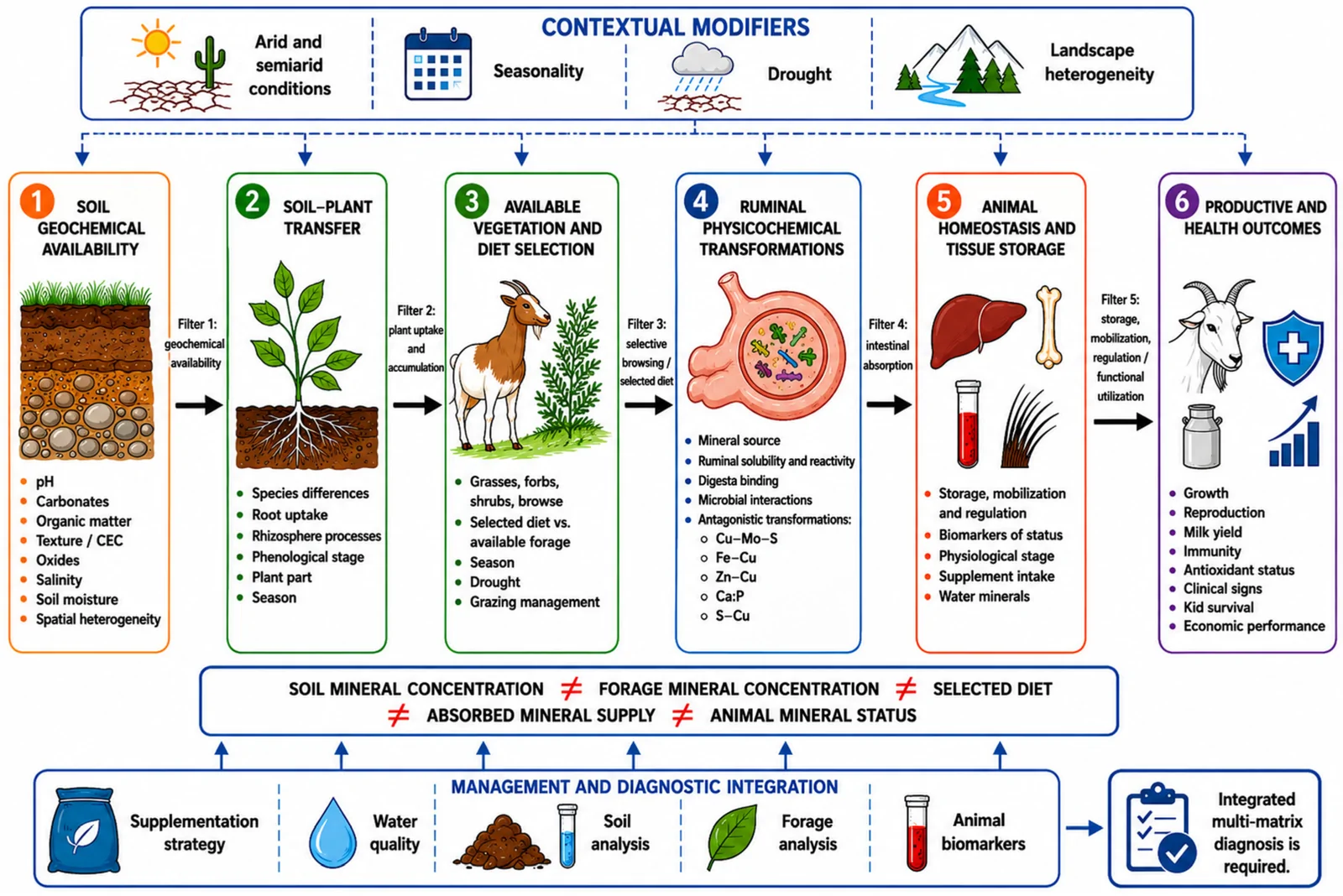
Conceptual framework of soil–plant–animal mineral dynamics in extensive goat production systems. The framework comprises six sequential stages linked by five functional filters: (1) soil geochemical availability, (2) soil–plant transfer, (3) available vegetation and diet selection, (4) ruminal physicochemical transformations and intestinal absorption, (5) animal homeostasis and tissue storage, and (6) productive and health outcomes. Contextual modifiers such as arid and semiarid conditions, seasonality, drought, and landscape heterogeneity act across the continuum. Key mineral interactions, including Cu–Mo–S, Fe–Cu, Zn–Cu, Ca:P, and S–Cu, can modify mineral solubility, ruminal transformations, intestinal absorption, and tissue status. Because soil mineral concentration does not directly predict forage mineral concentration, selected diet, bioavailable mineral supply, or animal mineral status, integrated multi-matrix diagnosis combining soil, forage, water, supplementation, and animal biomarkers is required. Arrows indicate the direction of the conceptual relationships among the components of the framework, whereas the colors are used only to visually distinguish the different stages and elements and do not represent levels of evidence or risk.

This complexity is particularly relevant in arid and semiarid systems, where heterogeneous soils, seasonal vegetation, selective browsing, and variable supplementation modify mineral exposure across time and space. Because goats can shift their intake among plant species and plant parts according to forage availability, season, and physiological stage, mineral intake cannot be inferred solely from soil or average forage composition [1,19]. The integrated framework developed here organizes these processes as sequential functional filters, as described in Section 7.2 and summarized in Figure 1.

### 7.2. Sequential Filters in the Soil–Plant–Animal Continuum

The proposed framework conceptualizes mineral transfer as a sequence of interconnected physiological and environmental filters, as summarized in Figure 1.

The first filter is geochemical availability in soil: Total mineral concentrations do not necessarily represent plant-available fractions because pH, carbonates, organic matter, texture, cation exchange capacity, oxides, salinity, and soil moisture influence mineral solubility and mobility. This is particularly important in alkaline, calcareous, and dryland soils, where micronutrients and phosphorus may be present but poorly available for plant uptake [10,13].

The second filter is soil–plant transfer: Plant species differ in their ability to absorb, translocate, and accumulate minerals, even when growing under similar soil conditions. In goat systems, this filter is critical because shrubs and browse species may supply relatively higher amounts of some minerals while providing lower amounts of others. Studies in semiarid and arid shrublands have shown that browse species may provide comparatively higher concentrations of calcium, magnesium, potassium, or sodium, whereas phosphorus, copper, zinc, iron, or manganese may occur at lower concentrations, with these patterns varying according to species and season [14,16].

The third filter is diet selection: Available forage does not necessarily represent the consumed diet because goats selectively choose plant species and plant parts. As a result, the mineral profile of the diet may differ substantially from the mineral profile of the vegetation available in the grazing area. This filter is especially important during drought or dry seasons, when goats may depend more heavily on woody species and persistent browse [1,2].

The fourth filter comprises ruminal physicochemical transformations and intestinal absorption. After ingestion, minerals are exposed to ruminal fermentation, microbial activity, pH variation, dissolution, precipitation, digesta binding, and antagonistic interactions that modify the chemical form and quantity of mineral reaching the lower gastrointestinal tract [22,23,24,52,53,54,55,56]. These ruminal processes are distinct from intestinal absorption, which determines the fraction transferred across the gastrointestinal epithelium into the animal and contributes to whole-animal mineral bioavailability and utilization [22,48,49].

The fifth filter is animal homeostasis and tissue storage: Once absorbed, minerals are transported, stored, mobilized, or excreted according to physiological regulation. Serum, plasma, whole blood, liver, bone, hair, and functional biomarkers reflect different biological compartments and time scales. Therefore, a multi-matrix animal assessment is more informative than a single biomarker when diagnosing mineral imbalance in extensive systems [30,54].

### 7.3. Interpreting Primary and Secondary Mineral Deficiencies

The proposed conceptual framework can support the interpretation of evidence consistent with primary or secondary mineral deficiencies, but it is not intended to classify these conditions through validated diagnostic thresholds or decision rules. A primary deficiency occurs when dietary mineral supply is insufficient to meet animal requirements. This may result from low soil availability, poor soil–plant transfer, low mineral concentration in preferred forage species, seasonal forage scarcity, or insufficient supplement intake. In semiarid goat systems, low dietary copper, zinc, and cobalt, together with high iron, have been reported under native vegetation-based conditions, indicating that primary deficiencies may occur even when animals have access to grazing resources [6].

A secondary deficiency occurs when a mineral is present in the diet but its absorption or utilization is reduced by antagonistic interactions. Copper deficiency is the clearest example. High dietary sulfur, molybdenum, or iron can reduce copper bioavailability, resulting in low tissue copper even when dietary copper appears adequate. In Yudong black goats, copper deficiency has been associated with soil and forage mineral patterns, high sulfur exposure, low blood and liver copper, hematological alterations, impaired immune and antioxidant status, and response to copper sulfate treatment [7].

This distinction has direct implications for supplementation. If a deficiency is primary, increasing the supply of the deficient mineral may be effective. If it is secondary, supplementation must also address the antagonistic context. For example, increasing copper alone may be insufficient when high sulfur, molybdenum, or iron continues to reduce copper absorption. Thus, mineral diagnosis should evaluate the deficient element together with interacting minerals, water composition, forage profile, and tissue reserves [8,22].

### 7.4. Spatial and Seasonal Dimensions of Mineral Risk

Mineral risk in extensive goat systems is spatially heterogeneous. Parent material, topography, erosion, sediment deposition, shrub cover, manure deposition, grazing pressure, water points, and management history can create mineral-rich and mineral-poor patches within the same production unit. Studies using spatial or correlation-based approaches show that soil and forage mineral concentrations can vary across regions and that relationships among soil, forage, and animal matrices are element-specific [13,37].

Seasonal variation adds another layer of complexity. Rainfall, drought, plant phenology, forage biomass, and botanical composition change throughout the year, modifying mineral uptake by plants and mineral intake by goats. In the Brazilian semiarid region, seasonal evaluation of goats using diet, serum, liver, and rib analyses showed that mineral interpretation depends on the time of year and on the biological matrix evaluated [6]. Similarly, diet composition in browsing goats can vary between seasons and physiological stages, affecting the mineral profile of the selected diet [1].

Because spatial and seasonal variation interact, one-time sampling may miss critical periods of mineral risk. Sampling only during the rainy season may underestimate deficiencies that appear during the dry season, while composite samples may obscure mineral patches. A robust diagnostic strategy should include repeated sampling during biologically relevant periods, such as the dry season, late pregnancy, early lactation, and growth [2,19].

### 7.5. Multi-Matrix Diagnosis and Interpretation

A practical soil–plant–animal framework should combine environmental, dietary, and animal matrices. At the soil level, relevant variables may include pH, carbonates, organic matter, texture, cation exchange capacity, salinity, and context-dependent operational soil-test indices such as Olsen-P and DTPA-extractable micronutrients [10,11,13,40,41,42,43,44]. At the forage level, mineral analysis should include the species and plant parts actually consumed by goats rather than only the most abundant vegetation. At the animal level, serum or plasma minerals should be interpreted together with liver reserves, hematology, antioxidant markers, immune indicators, productive performance, and clinical signs when possible [30,54].

For field application, sampling should be adapted to the heterogeneity of the production system. In extensive rangeland or shrubland systems, soil and vegetation sampling should account for contrasting soil, vegetation, and grazing-use areas rather than relying exclusively on whole-farm composite samples, while forage sampling should prioritize the plant species and plant parts actually selected by goats [1,13,14,19,37]. In semi-extensive or supplemented systems, assessment should additionally characterize basal forage, drinking-water mineral composition, supplement formulation, and actual supplement intake [8,22,62]. Across systems, animal sampling should account for physiological and productive stages, and repeated assessment should be considered during contrasting seasons or periods of increased mineral demand, particularly the dry season, late pregnancy, early lactation, and growth [2,6,30,54].

Water and supplements should also be included in the diagnostic framework. Drinking water may contribute sulfur, sodium, iron, calcium, magnesium, salinity-related ions, or other minerals that modify total intake or antagonistic risk. Mineral supplements may correct deficiencies, but their effect depends on formulation, mineral source, palatability, access, and actual intake. Therefore, supplement composition should not be evaluated only on paper; it should be interpreted in relation to basal diet, antagonists, and animal response [8,22].

Correlation analysis can help explore relationships among matrices, but it should not be treated as definitive proof of causality. In a soil–plant–animal study conducted in sheep, feed, fodder, and serum mineral concentrations were associated, whereas serum trace mineral concentrations did not correlate directly with soil [34]. Similarly, evidence from yaks showed complex and element-specific correlations among soil, forage, hair, and serum mineral concentrations across different regions [37]. These findings indicate that statistical relationships must be interpreted together with mineral chemistry, plant uptake, diet selection, ruminal physicochemical transformations, intestinal absorption, and animal physiology.

### 7.6. Decision-Making for Targeted Supplementation

The purpose of an integrated framework is to support more precise supplementation decisions. Generic mineral mixtures may fail when they do not match the local mineral profile of soil, forage, water, and animals. They may also be ineffective when the problem is a secondary deficiency caused by antagonists rather than a simple dietary shortage. Therefore, supplementation should be based on the identification of limiting minerals, antagonistic elements, and the physiological stage of the herd [6,8].

If phosphorus deficiency is suspected, diagnosis should integrate soil Olsen-P, forage phosphorus, Ca:P ratio, selected diet, growth, reproduction, and bone or serum indicators. If copper deficiency is suspected, copper should be evaluated together with molybdenum, sulfur, iron, water minerals, liver copper, hematological variables, immune markers, and antioxidant status. If zinc or cobalt deficiency is suspected, forage concentration, soil availability, serum or plasma indicators, growth, immune function, and supplement intake should be considered together [7,16].

Targeted supplementation should also be seasonal. During dry periods, goats may consume more shrubs and lower-quality forage, while during lactation or growth, mineral requirements increase. As a result, a mineral mixture adequate for maintenance during the rainy season may be insufficient during the dry season or early lactation. Region- and season-specific supplementation is therefore more consistent with the biology of extensive goat systems than static year-round supplementation [1,6].

At the farm level, the framework can be used to identify where mineral supply becomes limiting by evaluating three linked levels. First, representative grazing areas should be characterized for soil mineral availability; second, the plant species and plant parts actually consumed by goats should be analyzed, preferably across contrasting seasons; and third, mineral status should be assessed in representative animals using mineral-appropriate biomarkers [1,6,7,16,19,30,72]. Concordance among these levels can then guide management: low mineral availability in soil together with low concentrations in selected forage and low animal status supports a primary supply limitation, whereas apparently adequate dietary supply combined with low animal status should prompt investigation of mineral antagonisms or reduced bioavailability before supplementation is increased [7,8,27,32]. Once a targeted nutritional intervention is implemented, the relevant animal indicators and productive or health response should be reassessed to verify whether the identified limitation was corrected [6,7].

### 7.7. Proposed Conceptual Model

The proposed framework integrates environmental, dietary, and animal evaluation levels into a coherent system. Each specific matrix answers a distinct diagnostic question within the continuum. Soil analysis determines whether minerals are geochemically available for plant uptake. Forage analysis evaluates whether edible plants contain minerals at concentrations compatible with general requirements, while selected-diet analysis reveals whether goats actually consume those specific plants and plant fractions.

At the digestive level, assessment of ruminal solubility, reactivity, digesta binding, and antagonistic interactions characterizes the physicochemical processes that determine the amount and chemical form of minerals reaching the lower gastrointestinal tract, whereas evidence of intestinal absorption is required to determine their subsequent entry into the animal. Finally, animal tissue and functional biomarkers confirm whether minerals are effectively absorbed, stored, and utilized, whereas productive and health indicators establish the systemic consequences of any existing imbalance. Operationally, this framework is organized into six diagnostic levels: soil environment, forage and selected diet, water and supplements, ruminal transformations and intestinal absorption, animal biomarkers, and productive or health response. This comprehensive framework allows mineral disorders to be interpreted as system-level outcomes rather than isolated deficiencies, helping identify plausible stages at which mineral transfer may be constrained along the soil–plant–animal chain. The integrated diagnostic framework for mineral risk assessment in extensive goat systems is summarized in Table 7.

## 8. Knowledge Gaps and Future Research Directions

### 8.1. Limited Goat-Specific Evidence Under Extensive Conditions

Goat-specific evidence on mineral dynamics under extensive grazing and browsing conditions remains limited, as much of the available information derives from sheep, cattle, yaks, or mixed small-ruminant systems. Although these studies provide useful mechanistic evidence, extrapolation to goats should be cautious because of differences in browsing behavior, diet selectivity, plant-part selection, and exposure to plant secondary compounds [1,72]. Future research should therefore prioritize goat-specific field studies that incorporate local breeds, relevant physiological stages, seasonal sampling, and representative low-input production conditions [6,31].

### 8.2. Fragmented Evaluation of the Soil–Plant–Animal Continuum

Mineral studies in goats frequently assess soil, forage, or animal status separately, limiting identification of the stage at which mineral transfer is disrupted. Integrated designs that evaluate environmental, dietary, and animal matrices concurrently are therefore needed to distinguish limitations related to geochemical availability, soil–plant transfer, diet selection, antagonistic interactions, supplementation, or physiological demand [33,34,35,37,77]. Goat field evidence further indicates that combining environmental and dietary information with animal biomarkers and functional responses can reveal mechanisms that may remain undetected in single-matrix evaluations [7].

### 8.3. Insufficient Characterization of Geochemical Availability

Overreliance on total soil mineral concentrations remains an important limitation because total pools do not necessarily reflect plant-available fractions, particularly in alkaline, calcareous, and semiarid soils where mineral availability is strongly influenced by soil physicochemical properties [10,11]. Future studies in extensive goat systems should therefore incorporate operational indicators of mineral availability, including DTPA-extractable Zn, Cu, Fe, and Mn and Olsen-P for plant-available P, together with appropriate consideration of spatial heterogeneity [9,13]. Such measurements would provide a more informative basis for identifying geochemical constraints on forage mineral supply.

### 8.4. Weak Distinction Between Available Forage and Selected Diet

Available forage should not be assumed to represent the diet consumed by goats because selective browsing varies with plant availability, season, physiological stage, and forage characteristics. Consequently, the botanical and mineral composition of the selected diet may differ substantially from that of the vegetation available in the grazing area [1,19]. Future studies should clearly distinguish available vegetation from selected diet and link mineral analysis to appropriate botanical diet-assessment methods. Species- and plant-part-specific sampling is particularly important in heterogeneous arid and semiarid browse systems, where mineral composition varies among forage resources and seasons [14,15,16].

### 8.5. Limited Goat-Specific Evidence on Ruminal Physicochemical Transformations and Intestinal Absorption

Goat-specific evidence separately characterizing ruminal mineral transformations and subsequent intestinal absorption remains limited, as much of the available information derives from other ruminants or in vitro models [22,23,24,48,49,52,53,54,55,56]. Future studies should distinguish ruminal solubility, reactivity, digesta binding, and antagonistic interactions from intestinal absorption and whole-animal mineral utilization under diets representative of extensive goat production systems [8,26].

### 8.6. Underrepresentation of Water and Supplement Intake

Water and mineral supplement intake remain undercharacterized in extensive production systems despite their potential contribution to mineral exposure. Drinking water can provide relevant amounts of S and Fe and should therefore be considered when investigating Cu antagonism or unexplained mineral imbalances [62]. Future goat studies should routinely characterize water mineral composition and quantify actual supplement intake, particularly when free-choice mineral mixtures are used. Evidence from semiarid sheep indicates that area-specific mineral supplementation can improve serum trace-mineral status and growth [34]; however, comparable goat-specific studies are needed to establish locally appropriate supplementation strategies.

### 8.7. Lack of Standardized Biomarker Interpretation

Interpretation of mineral biomarkers remains inconsistent across studies because biological matrices reflect different physiological compartments and time scales, while analytical methods, reference intervals, and diagnostic thresholds also vary [54,70]. Future research should prioritize standardized, mineral-specific biomarker panels for goats, with matrix selection guided by the diagnostic objective and physiological context. Circulating mineral concentrations should not automatically be considered equivalent to tissue reserves, and alternative matrices should be validated against established indicators. Comparative evidence in sheep and goats confirms matrix-dependent element biodistribution and indicates that hair should not be considered interchangeable with blood, plasma, or serum [30].

### 8.8. Insufficient Linkage with Productive and Health Outcomes

Mineral studies should extend beyond concentration data to determine whether mineral imbalances are associated with biologically relevant outcomes. Future goat studies should therefore integrate mineral status with productive, reproductive, immune, antioxidant, and clinical indicators, particularly when evaluating subclinical imbalances. Goat-specific evidence demonstrates that altered Cu status can affect hematology, antioxidant capacity, immune responses, and tissue reserves even when short-term growth responses are limited or absent [7,8].

### 8.9. Need for Spatial, Seasonal, and Predictive Approaches

Mineral risk in extensive goat systems varies across locations and seasons; consequently, single-time-point or highly aggregated sampling may fail to identify periods or areas of mineral imbalance. Repeated seasonal and spatially explicit sampling should therefore be incorporated into field studies [6,37]. Future research should also develop region-specific predictive and decision-support approaches that integrate environmental, forage, and animal data. Evidence from multi-matrix studies indicates that relationships among soil, forage, and animal mineral concentrations are complex and context-dependent, supporting the need for models validated under local production conditions [33,34,37]. The main knowledge gaps and future research priorities identified in this review are summarized in Table 8.

## 9. Strengths and Limitations of the Review

### 9.1. Strengths

- The main strength of this review is its integrative perspective. Rather than treating mineral disorders in goats as isolated dietary deficiencies or animal-level abnormalities, this work organizes the available evidence within a holistic soil–plant–animal continuum. This approach allows mineral status to be interpreted as the outcome of sequential, interacting processes—spanning geochemical availability, soil–plant transfer, selected diet, ruminal physicochemical transformations, intestinal absorption, tissue storage, functional biomarkers, and productive or health responses.
- A second strength is the explicit focus on extensive goat production systems, particularly those located in drylands, rangelands, shrublands, and semiarid agroecosystems. These environments are characterized by heterogeneous soils, seasonal forage availability, selective browsing, variable water quality, and inconsistent supplement intake, making mineral diagnosis far more complex than in controlled feeding operations or uniform pasture systems. By emphasizing this challenging context, the review directly addresses a production context in which subclinical deficiencies, secondary mineral antagonisms, and seasonal mineral risks are highly prevalent.
- Another strength is the clear distinction maintained between available forage and the selected diet. In goats, this distinction is crucial because the overall mineral composition of the vegetation present in a landscape differs substantially from the mineral profile of the specific plant species and parts consumed. This review highlights the necessity of integrating vegetation surveys with botanical diet composition, browsing behavior, and seasonal plant availability.
- Furthermore, this review explicitly distinguishes ruminal physicochemical transformations from subsequent intestinal absorption and whole-animal bioavailability. This is particularly relevant for trace elements, where mineral source, ruminal solubility and reactivity, microbial activity, and antagonistic interactions can modify the amount and chemical form of minerals reaching the intestine and thereby influence subsequent absorption. The detailed discussion of the copper–molybdenum–sulfur interaction, mineral sources, and rumen solubility strengthens the systemic interpretation of secondary deficiencies in browsing ruminants.
- Additionally, the inclusion of animal biomarkers as the final integrative level of diagnosis represents a key asset. The review simultaneously considers serum, plasma, whole blood, liver, bone, hair, hematological indicators, immune markers, antioxidant status, clinical signs, and productive responses. This multi-matrix perspective acknowledges that no single biological substrate can fully represent the status of all elements or reflect every physiological time scale.
- Finally, this work moves beyond descriptive synthesis by providing a structured conceptual framework and a targeted research agenda. By identifying precise gaps related to goat-specific evidence, operational geochemical availability, water and supplement tracking, biomarker standardization, and predictive modeling, the review provides an interpretive framework to guide integrated mineral assessment and future validation in extensive goat production systems.

### 9.2. Limitations

Despite its comprehensive approach, several limitations inherent to the nature of the available literature must be acknowledged:

- High heterogeneity of the evidence base: The compiled literature varies widely in terms of animal breeds, production environments, geographical regions, analyzed elements, analytical methods, and reported outcome variables. This extreme heterogeneity prevented a quantitative synthesis or meta-analysis, justifying the use of a critical narrative approach. Consequently, this review cannot provide pooled effect estimates or standardized quantitative comparisons across studies.
- Scarcity of goat-specific data: A reference-level audit of the 75 sources retained in the structured eligibility matrix showed that 13 references (17.3%) were specifically focused on goats [1,4,6,7,8,15,16,17,31,72,73,74,75], whereas 14 references (18.7%) addressed mixed small-ruminant systems or explicitly included goats together with sheep or other small ruminants [2,3,14,19,20,21,30,32,33,46,50,51,69,76]. Fifteen references (20.0%) were based on other ruminant species without goat-specific data, including sheep, cattle, and yaks [26,28,34,37,52,53,54,55,56,57,58,61,62,68,71,77]. The remaining 33 references (44.0%) comprised species-general ruminant or livestock literature, forage or mechanistic/in vitro evidence (*n* = 21), and soil-, plant-, or methodology-focused sources without a species-specific animal dataset (*n* = 12). Thus, only 13 of 75 references were strictly goat-specific, while 27 of 75 (36.0%) were either goat-specific or goat-inclusive. The two additional goat-specific microbiome studies incorporated during peer-review revision were treated as contextual mechanistic evidence and were therefore not included in these denominator-based counts. This distribution quantifies the extent to which interpretation of mineral dynamics in extensive goat systems necessarily relies on comparative or extrapolated evidence and reinforces the need for goat-specific, longitudinal, and multi-matrix studies.
- Fragmentation of the continuum in primary studies: Most published studies evaluate soil, forage, or animal mineral status as isolated components. As a result, certain integrative conclusions in this review are built by bridging complementary separate studies rather than by reviewing single, long-term experiments that monitored the complete pathway from soil geochemistry to animal performance.
- Lack of standardized biomarker interpretation: Analytical matrices and diagnostic thresholds are inconsistent across historical and modern literature. Serum, plasma, liver, bone, and hair reflect entirely different biological compartments and metabolic time scales. This lack of standardization limits direct comparisons between regions, particularly for trace elements like copper, zinc, selenium, and manganese, where circulating blood values do not always mirror tissue reserves or functional enzyme activity.
- Underrepresentation of spatial and seasonal dynamics: Many field studies rely on one-time sampling or highly aggregated composite samples that fail to capture the real-world heterogeneity of extensive grazing landscapes. This may limit the detection of mineral risks, as soil availability, botanical preferences, water quality, and animal demands fluctuate drastically between rainy and dry seasons.
- Inconsistent tracking of water and supplement inputs: Drinking water composition and actual individual supplement consumption are chronically underreported in extensive research trials. In semiarid environments, water often acts as a major source of minerals or antagonists like sulfur and iron, while free-choice supplementation rarely guarantees uniform intake. The lack of precise data on these two variables restricts definitive causal interpretations of field imbalances.
- Geographical bias in database accessibility: While this review prioritized international, peer-reviewed literature, relevant research from underrepresented or remote dryland regions is frequently absent from major indexing databases or only accessible via regional, non-English journals. This may limit the global representativeness of the evidence base regarding low-input, marginalized goat production systems.

### 9.3. Interpretation of the Review Limitations

Taken together, these limitations define the boundaries of the current evidence base and support cautious, context-specific interpretation of mineral dynamics in extensive goat production systems. Accordingly, the proposed soil–plant–animal framework should be regarded as a conceptual structure for organizing available evidence and guiding future validation rather than as a validated diagnostic algorithm.

## 10. Conclusions

Mineral nutrition in extensive goat production systems is a dynamic soil–plant–animal process in which mineral transfer is modified at successive environmental, dietary, digestive, and physiological stages. Soil geochemical availability does not necessarily predict forage mineral composition or animal mineral status, while the mineral contribution of forage and browse is further influenced by plant species, plant part, phenological stage, season, and selective feeding behavior.

Mineral imbalances may arise from inadequate supply, increased physiological demand, or antagonistic interactions that impair absorption and utilization, with the Cu–Mo–S system representing a major example of secondary deficiency. Because environmental and dietary risks do not necessarily translate directly into animal mineral status, diagnosis should integrate mineral-specific biomarkers and complementary matrices appropriate to the production context.

Future research should prioritize goat-specific, seasonal, and spatially explicit studies that integrate geochemical availability, selected diet, water and supplement inputs, ruminal physicochemical transformations, intestinal absorption, and animal-level bioavailability, validated biomarkers, and functional outcomes. Such approaches should improve causal interpretation, support targeted supplementation, and strengthen sustainable mineral management in extensive goat production systems.

## Figures and Tables

**Table 1:** Eligibility criteria used for study selection.

Criterion	Inclusion Criteria	Exclusion Criteria
**Publication type**	Peer-reviewed original articles, reviews, systematic reviews, methodological studies, and regional case studies	Non-verifiable documents, incomplete abstracts, duplicated records, or sources lacking bibliographic information
**Publication period**	1 January 2019–29 June 2026; earlier studies retained only when foundational, methodologically relevant, or important for underrepresented regions or production systems	Earlier studies without foundational, methodological, regional, or production-system relevance
**Language**	Full-text articles available in English or Spanish	Articles in other languages when a complete English or Spanish full-text version or translation was not available for eligibility assessment
**Species**	Goats, sheep, small ruminants, and other ruminants when mechanistically relevant	Non-ruminants without transferable relevance
**Production system**	Extensive, semi-extensive, grazing, browsing, rangeland, dryland, arid, semiarid, shrubland, or silvopastoral systems	Fully intensive systems without relevance to soil, forage, grazing, or mineral bioavailability
**Environmental matrices**	Soil, forage, browse, pasture, shrubs, water, diet, or supplements	Studies without environmental or dietary relevance
**Animal matrices**	Serum, plasma, blood, liver, bone, hair, milk, rumen fluid, feces, hematology, immunity, antioxidant status, productivity, or clinical signs	Studies without mineral-related animal indicators
**Minerals**	Macro- and trace elements relevant to the soil–plant–animal continuum. Mechanistic synthesis focused primarily on mineral systems for which sufficient and transferable evidence was available, particularly Cu–Mo–S interactions, P, and Zn; other elements, including Se, Co, Fe, Mn, Ca, Mg, Na, and K, were retained when they contributed relevant forage, animal-status, biomarker, or comparative evidence.	Studies unrelated to mineral nutrition
**Review relevance**	Evidence contributing to soil–plant–animal mineral dynamics, bioavailability, mineral imbalance, or methodological integration	Isolated findings not interpretable within the review framework

**Table 2:** Thematic search strategy used for literature identification.

Thematic Domain	Example Search String
Animal mineral status	(goat* OR caprine OR “small ruminant*”) AND (“mineral status” OR “trace element*” OR “mineral profile”) AND (serum OR plasma OR blood OR liver OR hair) AND (grazing OR extensive OR rangeland OR pasture)
Soil geochemical availability	(“soil mineral availability” OR “geochemical availability” OR “DTPA-extractable” OR “Olsen phosphorus”) AND (calcareous OR alkaline OR carbonate OR arid OR semiarid) AND (zinc OR copper OR iron OR manganese OR phosphorus)
Forage and soil–plant transfer	(“forage mineral composition” OR “browse species” OR “shrub mineral content”) AND (goat* OR sheep OR “small ruminant*”) AND (arid OR semiarid OR rangeland OR shrubland)
Diet selection	(goat* OR sheep OR “small ruminant*”) AND (“diet selection” OR browsing OR “botanical composition”) AND (season* OR drought OR rangeland OR semiarid)
Ruminal solubility, antagonisms, and mineral bioavailability	(“rumen solubility” OR “trace mineral bioavailability” OR “ruminal solubility”) AND (copper OR zinc OR manganese OR molybdenum OR sulfur OR thiomolybdate*) AND (goat* OR sheep OR ruminant*)
Integrated models and drylands	(“soil-plant-animal” OR “soil forage animal” OR continuum) AND (mineral* OR “trace element*”) AND (goat* OR sheep OR ruminant*) AND (dryland* OR arid OR semiarid OR rangeland)

Note: The asterisk (*) indicates the truncation wildcard used to retrieve word variants in database searches.

**Table 3:** Main soil factors influencing mineral availability and their relevance for extensive goat production systems.

Soil Factor	Main Effect on Mineral Availability	Minerals Most Affected	Relevance for Goat Systems
pH	Regulates solubility, precipitation, adsorption, and mobility of mineral elements	Fe, Zn, Mn, Cu, P	High pH may reduce micronutrient availability to forage plants
Carbonates	Promote alkaline conditions and precipitation or sorption reactions	P, Zn, Fe, Mn, Cu	Common in drylands; may reduce plant-available minerals despite adequate total content
Organic matter	Enhances nutrient cycling, water retention, microbial activity, and complexation	P, Zn, Cu, Fe, Mn	Low organic matter may limit mineral transfer to forage in drylands
Texture and clay content	Regulate adsorption, desorption, water retention, and cation exchange	Zn, P, Cu, Mn, Fe	May retain nutrients but also reduce availability depending on mineral form
Fe and Mn oxides	Adsorb or co-precipitate trace elements and phosphorus	Zn, P, Cu, Mn	Can restrict plant uptake of micronutrients
Cation exchange capacity	Determines retention and exchange of nutrient cations	Ca, Mg, K, Na, Cu, Zn, Mn	Influences exchangeable mineral pools available to plants
Soil moisture	Controls diffusion, microbial activity, root function, and seasonal nutrient cycling	P, Zn, Fe, Mn, Cu	Drought may reduce mineral uptake and alter forage mineral composition
Spatial heterogeneity	Produces local variation in mineral availability across the landscape	All minerals	Creates mineral patches that may influence forage composition and goat exposure

**Table 4:** Key forage-related factors influencing mineral intake in extensive goat systems.

Forage-Related Factor	Main Influence on Mineral Nutrition	Practical Implication
Plant species	Determines mineral uptake, translocation, and tissue accumulation	Mineral analysis should be species-specific when possible
Plant functional group	Grasses, forbs, shrubs, and trees differ in mineral profiles	Browse and herbaceous forage should be interpreted separately
Plant part	Leaves, stems, pods, flowers, fruits, and shoots differ in mineral concentration	Sampling should reflect the parts actually consumed by goats
Season	Alters forage availability, mineral uptake, and botanical composition	Wet and dry seasons should be sampled separately
Phenological stage	Affects mineral dilution, accumulation, and senescence	Mineral adequacy may change during plant development
Selected diet	Determines actual mineral intake more directly than available biomass	Botanical diet composition should complement forage analysis
Grazing management	Affects access to plant species, patches, and mineral resources	Stocking rate, herding, and paddock use can modify mineral exposure
Secondary compounds	May influence intake, digestibility, rumen function, and mineral utilization	Mineral interpretation should consider tannins and other plant compounds
Soil–plant transfer	Links soil availability with plant mineral composition	Soil and forage should be interpreted jointly
Supplementation context	Modifies mineral intake and potential antagonisms	Supplement formulation should reflect local forage mineral profiles
Quantitative mineral benchmarking	Across the summary values reported in the two field studies, sampled browse/shrub material contained approximately 1.3–2.29 g P/kg DM, 4.20–6.4 mg Cu/kg DM, and 2.67–17.7 mg Zn/kg DM [14,16].	For comparison, NRC [46] data for a 50 kg dairy doe in early lactation nursing twins yield approximate whole-diet concentration equivalents of 2.97 g P/kg DM and 43.7 mg Zn/kg DM, while NRC separately recommends 15 mg Cu/kg diet DM for lactating goats. Because the field values were measured in sampled leaves or shrub parts rather than in the animal’s complete consumed diet, these comparisons are illustrative and should not be interpreted as direct assessments of dietary adequacy.

**Table 5:** Main mineral interactions relevant to extensive goat production systems.

Interaction	Mechanism	Potential Consequence	Diagnostic Implication	Evidence Level in This Review
Cu–Mo–S	Formation of thiomolybdates in the rumen reduces Cu absorption	Secondary Cu deficiency, reduced liver Cu, impaired immunity	Evaluate Cu together with Mo, S, Fe, water minerals, and liver Cu	High—Consistent mechanistic and experimental evidence, including direct goat studies [7,8,27,28,29,49,57,58,59]
Fe–Cu	High Fe may interfere with Cu utilization and metabolism	Reduced Cu status, especially when Cu intake is marginal	Interpret high-Fe diets cautiously in low-Cu systems	Moderate—Supported by ruminant mineral-interaction evidence and field observations, but direct goat-specific experimental validation remains limited [6,49,59]
Zn–Cu	Excessive or imbalanced Zn and Cu may affect absorption or metabolism	Altered trace mineral balance	Avoid interpreting Zn and Cu independently	Limited—Biologically plausible and supported by trace-mineral and ruminal studies, but direct evidence for the interaction in extensively managed goats is scarce [49,60,61]
Ca–P	Imbalanced Ca:P ratio affects P utilization and skeletal metabolism	Poor growth, reproductive problems, bone disorders	Evaluate forage Ca and P together with animal indicators	Moderate—Well-established nutritional interaction with relevant forage and goat-requirement evidence, although relatively few studies directly evaluate the interaction under extensive goat conditions [14,16,46]
S–Cu	High sulfur may reduce Cu absorption, with or without high Mo	Increased risk of secondary Cu deficiency	Include forage and water S in Cu deficiency investigations	High—Strong mechanistic evidence and direct relevance to secondary Cu deficiency, including goat-specific evidence [7,8,27,57,59,62]
Mineral source–rumen solubility	Mineral sources differ in dissolution, reactivity, microbial association, and binding within the rumen, thereby altering the amount and chemical form reaching the lower gastrointestinal tract [23,24,52,53,54,55,56]	Different quantities and chemical forms may reach the intestine; ruminal solubility alone does not demonstrate intestinal absorption or whole-animal bioavailability [22,23,24,48,49]	Interpret ruminal solubility and reactivity separately from intestinal absorption and whole-animal relative bioavailability	High—Consistently supported by experimental and in vitro ruminant studies across multiple mineral sources [22,23,24,25,52,53,54,55,56]
Trace minerals–rumen microbiota	Cu, Zn, Mn, Fe, Co, and Se may affect microbial growth, fermentation, and fiber digestion	Altered fermentation, digestibility, methane production, or microbial diversity	Interpret mineral supplementation together with rumen function	Moderate—Supported by several experimental, in vitro, cattle, sheep, and small-ruminant models, with limited direct validation in extensive goats [49,61,65,66,67,68,69]

Note: Evidence levels are qualitative and specific to the body of literature evaluated in this review rather than a formal certainty-of-evidence grading. High indicates consistent support from multiple independent mechanistic or experimental studies, including goat-specific evidence when available; Moderate indicates support from several relevant studies but limited direct validation in goats under extensive conditions; and Limited indicates predominantly indirect, mechanistic, or extrapolated evidence with scarce direct validation in extensive goat systems.

**Table 6:** Main animal biomarkers for evaluating mineral status in goats.

Biomarker or Matrix	Main Diagnostic Value	Advantages	Limitations
Serum/plasma	Circulating mineral status	Practical, minimally invasive, useful for herd screening	May be homeostatically regulated; not always reflective of tissue reserves
Whole blood	Mineral distribution in cellular and plasma fractions	Useful for comparative biomonitoring and some trace elements	Matrix-specific interpretation required; not interchangeable with serum/plasma
Liver	Tissue reserves, especially Cu and selected trace elements	Strong indicator of long-term status for some minerals	Invasive; requires biopsy or postmortem sampling
Bone/rib	Long-term Ca and P status	Useful for chronic macromineral assessment	Invasive; less practical for routine field diagnosis
Hair	Complementary biomonitoring and longer-term exposure	Non-invasive, easy to store and collect	External contamination, pigmentation, sampling site, and weak equivalence with blood
Tears/saliva	Potential alternative matrices for selected elements	Minimally invasive and useful for exploratory biomonitoring	Requires validation against established matrices
Hematology	Functional effects of mineral imbalance	Useful for anemia and systemic effects	Not mineral-specific
Antioxidant markers	Oxidative stress and enzyme-related mineral function	Useful for Cu, Se, Zn, and Mn-related disorders	Requires laboratory capacity and careful interpretation
Immune markers	Biological relevance of trace mineral imbalance	Detects subclinical functional impairment	Influenced by infection, stress, parasites, and disease
Productive indicators	Growth, reproduction, milk, kid survival, herd performance	Direct practical relevance	Nonspecific and affected by multiple non-mineral factors

**Table 7:** Integrated diagnostic framework for mineral risk assessment in extensive goat systems.

Diagnostic Level	Main Indicators	Main Diagnostic Question	Interpretation
Soil environment	pH, carbonates, organic matter, texture, CEC, salinity, Olsen-P, DTPA-extractable trace elements	Do soil properties and soil-test indices indicate potential constraints on plant mineral supply?	Identifies potential edaphic constraints; soil-test indices are context dependent and do not directly measure plant or animal bioavailability.
Forage and selected diet	Mineral profile of edible species and plant parts; botanical composition; seasonal selection	What mineral supply is available and actually consumed?	Links soil–plant transfer with real dietary exposure in selective browsers.
Water and supplements	Water minerals, sulfur, salinity, supplement formula, source, ratios, palatability, and intake	Do additional inputs correct deficiencies or add antagonists?	Clarifies external mineral inputs and effectiveness of supplementation.
Ruminal transformations and intestinal absorption	Mineral source, ruminal solubility and reactivity, digesta binding, antagonistic interactions, and evidence of intestinal absorption	What chemical forms and quantities leave the rumen, and what fraction is subsequently absorbed?	Distinguishes ruminal physicochemical behavior from intestinal absorption and whole-animal bioavailability; ruminal solubility alone should not be interpreted as evidence of absorption or utilization.
Animal biomarkers	Serum, plasma, blood, liver, bone, hair, hematology, antioxidant enzymes, immune markers, and ceruloplasmin	Are minerals absorbed, stored, and functional?	Confirms biological imbalance according to mineral, matrix, and exposure time scale.
Productive and health response	Growth, reproduction, milk yield, kid survival, disease susceptibility, clinical signs, and response to supplementation	Does mineral imbalance affect performance or health?	Provides practical endpoints; nonspecific without environmental and laboratory confirmation.

**Table 8:** Main knowledge gaps and future research priorities.

Knowledge Gap	Current Limitation	Future Research Priority	Evidence Supporting the Gap
Goat-specific evidence	Much evidence comes from sheep, cattle, yaks, or mixed ruminant systems	Conduct field studies specifically in goats under extensive grazing and browsing conditions	High—Quantitatively documented in this review: only 13/75 references were strictly goat-specific and 27/75 were goat-specific or goat-inclusive
Soil–plant–animal integration	Soil, forage, diet, and animal data are often studied separately	Design multi-matrix studies including soil, forage, selected diet, water, supplements, and biomarkers	High—Multiple studies evaluate only partial components of the continuum, whereas relatively few integrate environmental, dietary, and animal matrices [7,33,34,35,37,77]
Geochemical availability	Total soil minerals are often overemphasized	Include DTPA-extractable trace elements, Olsen-P, pH, carbonates, organic matter, CEC, and salinity	High—Consistent soil evidence shows that total mineral concentration does not adequately represent plant-available fractions, particularly in alkaline and calcareous soils [9,10,11,13]
Available forage vs. selected diet	Available vegetation is often assumed to equal consumed diet	Evaluate botanical composition and plant parts actually selected by goats using validated botanical diet-assessment methods.	High—Direct goat and small-ruminant evidence consistently shows that available vegetation may differ from the diet actually selected [1,14,15,16,19]
Ruminal transformations and mineral bioavailability	Goat-specific evidence under field challenge is limited	Study mineral chemical source, ruminal solubility and reactivity, microbial response, antagonistic interactions, intestinal absorption, and animal-level bioavailability in goats	High—The gap is clearly documented because much of the available evidence derives from other ruminants or in vitro models, with limited direct goat-specific validation [8,22,23,26]
Water and supplements	Mineral contribution from water and actual supplement intake are often ignored	Measure water minerals and quantify individual supplement intake under field conditions	Moderate—Their biological relevance is supported, but direct quantification of water mineral exposure and individual supplement intake under extensive goat conditions remains limited [34,62]
Biomarker interpretation	Diagnostic matrices and reference thresholds are inconsistent	Develop validated mineral-specific biomarker panels and reference ranges for goats	High—Multiple sources demonstrate matrix-specific differences, inconsistent diagnostic thresholds, and limited interchangeability among biomarkers [30,54,70]
Productive relevance	Mineral concentrations are not always linked to biological outcomes	Connect mineral status with growth, reproduction, immunity, oxidative status, health, and economic performance	Moderate—Goat-specific studies demonstrate functional consequences of mineral imbalance, but direct linkage with long-term productive and economic outcomes remains limited [7,8]
Spatial and seasonal variation	One-time or composite sampling may hide localized mineral risks	Use repeated seasonal sampling, georeferenced designs, and landscape-level geostatistics	Moderate—Seasonal and spatial variability is supported by field and multi-matrix studies, but longitudinal and spatially explicit goat datasets remain scarce [6,33,34,37]
Decision support	Most field frameworks remain purely descriptive	Develop region-specific targeted supplementation models and predictive mineral-risk tools	Limited—The need is mainly inferred from the scarcity of validated predictive or region-specific decision-support models in the reviewed literature; available evidence is primarily descriptive or correlation-based [33,34,37]

Note: Evidence levels refer to how clearly each knowledge gap is documented within the literature evaluated in this review and do not constitute a formal certainty-of-evidence grading. High indicates that the gap is consistently supported by multiple independent studies or by quantitative evidence within the review; Moderate indicates that the gap is supported by relevant evidence but direct goat-specific or field validation remains incomplete; and Limited indicates that the gap is inferred mainly from sparse, indirect, or emerging evidence.

## Data Availability

All data supporting this review are contained within the article and Supplementary Table S1.

## Supplementary Materials

The following supporting information can be downloaded at: https://www.mdpi.com/article/10.3390/ani16172790/s1; Table S1: Study-level extraction and evidence matrix for the literature included in the structured synthesis [1,2,3,4,5,6,7,8,9,10,11,12,13,14,15,16,17,18,19,20,21,22,23,24,25,26,27,28,29,30,31,32,33,34,35,36,37,38,39,40,41,42,43,44,45,46,47,48,49,50,51,52,53,54,55,56,57,58,59,60,61,62,63,64,65,66,67,68,69,70,71,72,73,74,75,76,77], including the methodological and evidence-base summary, contextual additions incorporated during peer-review revision and excluded from the denominator-based 75-reference counts, and the corresponding codebook.

## Abbreviations

The following abbreviations are used in this manuscript:

Ca	Calcium
CEC	Cation exchange capacity
Co	Cobalt
Cu	Copper
DOI	Digital object identifier
DM	Dry matter
DTPA	Diethylenetriaminepentaacetic acid
Fe	Iron
K	Potassium
Mg	Magnesium
Mn	Manganese
Mo	Molybdenum
Na	Sodium
Olsen-P	Olsen-extractable phosphorus
P	Phosphorus
PRISMA	Preferred Reporting Items for Systematic Reviews and Meta-Analyses
PRISMA-S	PRISMA extension for reporting literature searches
S	Sulfur
Se	Selenium
URL	Uniform resource locator
Zn	Zinc
